# Effects of hesperidin supplementation on inflammation and oxidative stress in overweight or obese individuals: a systematic review and meta-analysis of randomized controlled trials

**DOI:** 10.3389/fnut.2026.1871474

**Published:** 2026-06-26

**Authors:** Kun Zhu, Wen Li, Tingting Liao, Jianlong Guo, Tingwei Huang, Degui Chang, Yaodong You

**Affiliations:** Hospital of Chengdu University of Traditional Chinese Medicine, Chengdu, Sichuan, China

**Keywords:** citrus matrix, hesperidin, inflammation, meta-analysis, obesity, overweight, oxidative stress

## Abstract

This study aimed to evaluate, through a systematic review and meta-analysis of randomized controlled trials (RCTs), the effects of hesperidin and hesperidin-containing citrus interventions on biomarkers of inflammation, oxidative stress, and endothelial function in overweight or obese individuals. Two authors independently searched PubMed, Web of Science, Embase, Scopus, and the Cochrane Library from inception to April 4, 2026. Methodological quality was assessed using the Cochrane Risk of Bias 2 tool (RoB 2), and certainty of evidence was evaluated with the Grading of Recommendations Assessment, Development and Evaluation (GRADE) approach. Heterogeneity was assessed using Cochran's *Q* test and the *I*-square (*I*^2^) statistic, and fixed- or random-effects models were applied as appropriate. Most continuous outcomes were pooled as mean differences (MDs) with 95% confidence intervals (CIs), whereas total antioxidant capacity (TAC) was pooled as standardized mean differences (SMDs; Hedges' *g*) due to inconsistent assay methods. Sixteen RCTs involving 845 participants were included. Compared with placebo or control, hesperidin-related interventions reduced C-reactive protein/high-sensitivity C-reactive protein (CRP/hsCRP) (MD: −0.43 mg/L, 95% CI: −0.68, −0.17, *P* = 0.001; GRADE = moderate), tumor necrosis factor-alpha (TNF-α) (MD: −2.84 pg/mL, 95% CI: −4.81, −0.86, *P* = 0.005; GRADE = very low), and vascular cell adhesion molecule-1 (VCAM-1) (MD: −27.16 ng/mL, 95% CI: −50.13, −4.19, *P* = 0.020; GRADE = very low), with no significant effects on interleukin-6 (IL-6), TAC, or intercellular adhesion molecule-1 (ICAM-1). Malondialdehyde (MDA) and superoxide dismutase (SOD) were reported narratively in single studies. Current randomized evidence suggests that hesperidin-related interventions are associated with a modest reduction in CRP/hsCRP, representing the most consistent biomarker signal to date. Evidence for TNF-α and VCAM-1 remains very low in certainty, and findings for IL-6, oxidative stress markers, and ICAM-1 remain inconclusive. Marked between-study differences in intervention matrix, design structure, and reporting methods preclude dose- or matrix-specific recommendations at present. Further high-quality randomized trials with longer follow-up, standardized biomarker assessment, and better characterization of formulations are needed.

## 1 Introduction

Overweight and obesity are not merely anthropometric states but are commonly accompanied by low-grade chronic inflammation, redox imbalance, and metabolic tissue stress. Adipose tissue expansion, immune cell infiltration, adipokine dysregulation, mitochondrial dysfunction, and increased reactive oxygen species generation collectively promote metabolic and vascular abnormalities including insulin resistance, fatty liver, vascular endothelial activation, and atherosclerosis (1–4). These shared abnormalities provide a rationale for simultaneously assessing inflammatory and oxidative stress biomarkers, and make individuals with overweight or obesity a reasonable target population for evaluating nutrition-related interventions.

Inflammation and oxidative stress cannot be fully captured by a single indicator. C-reactive protein (CRP) or high-sensitivity C-reactive protein (hsCRP) are commonly reported downstream acute-phase markers with established clinical interpretation frameworks (5–7), while interleukin-6 (IL-6) and tumor necrosis factor-α (TNF-α) reflect more upstream cytokine signals (8); total antioxidant capacity (TAC) and malondialdehyde (MDA) provide oxidative stress information from the perspectives of overall antioxidant capacity and lipid peroxidation, respectively. Indicators such as intercellular adhesion molecule-1 (ICAM-1), vascular cell adhesion molecule-1 (VCAM-1), and superoxide dismutase (SOD) can additionally reflect vascular endothelial activation and antioxidant defense. Constructing an outcome hierarchy based on these complementary readouts better captures the pathological features associated with overweight and obesity than emphasizing a single inflammatory biomarker alone.

Individuals with overweight or obesity represent an important target for nutrition-based adjunctive strategies (9, 10). Hesperidin is a flavanone glycoside abundant in oranges and related citrus products and can be delivered as purified supplements or within citrus matrices such as orange juice, flavonoid-rich beverages, or whole fruit (11, 12). Experimental evidence suggests that hesperidin and its aglycone metabolite hesperetin may modulate inflammatory and oxidative pathways, providing biological plausibility for effects on inflammatory and oxidative stress biomarkers (13–15). However, oral exposure is shaped by intestinal deglycosylation, microbial metabolism, matrix co-components, bioavailability, and formulation design; therefore, clinical effects may differ across purified and food-based intervention forms.

Existing clinical reviews suggest that hesperidin or citrus-related interventions may affect selected inflammatory and oxidative stress biomarkers, but conclusions are inconsistent. Previous studies have varied substantially in population scope, intervention forms, and outcome hierarchies: a 2019 meta-analysis by Loizadeh et al. (16) including 6 studies found no effect of hesperidin on overall CRP or IL-6, while a later meta-analysis in healthy populations reported reductions in CRP, IL-6, and MDA (17); a recent adult meta-analysis found reductions in CRP/hsCRP and TNF-α but no overall benefit for IL-6 (18); broader cardiometabolic analyses showed selective rather than universal benefits (19); and an orange juice review suggested IL-6 reduction while hsCRP did not reach significance with very low evidence strength (20). These discrepancies indicate that existing evidence does not directly address the question in individuals with overweight or obesity, a population with high obesity-related inflammatory and oxidative stress burden. Therefore, when purified hesperidin and hesperidin-containing citrus matrices are considered together, it remains unclear whether oral hesperidin-related exposures can produce consistent changes in inflammatory and oxidative stress biomarkers in individuals with overweight or obesity. To address this, the present study evaluated the effects of hesperidin and hesperidin-containing citrus interventions on inflammatory and oxidative stress biomarkers in individuals with overweight or obesity through a systematic review and meta-analysis of randomized controlled trials (RCTs). CRP/hsCRP was evaluated as the primary poolable inflammatory outcome, while IL-6, TNF-α, TAC, MDA, and endothelial function-related supplementary indicators were also included, with exploratory assessment of whether intervention form, duration, dose, population characteristics, and lifestyle co-interventions might influence results.

## 2 Methods

### 2.1 Reporting standards and protocol

The design, conduct, and reporting of this systematic review and meta-analysis followed the Preferred Reporting Items for Systematic Reviews and Meta-Analyses (PRISMA) 2020 statement and its explanation and elaboration documents (21, 22) and the A MeaSurement Tool to Assess systematic Reviews 2 (AMSTAR 2) quality appraisal framework (23) to ensure methodological rigor and reporting completeness. The study protocol was also registered with the International Prospective Register of Systematic Reviews (PROSPERO) (Number = CRD420261358301).

### 2.2 Data sources and search strategy

We systematically searched PubMed, Web of Science, Embase, Scopus, and the Cochrane Library from inception to April 4, 2026, without language restrictions. The search strategy combined terms related to hesperidin, citrus flavonoids, orange juice, overweight or obesity, and RCTs. Because the review question concerned oral hesperidin-related exposures rather than a single commercial product, the search strategy allowed both purified supplement terms and citrus food matrix terms to enter screening. The complete search strategy is provided in Supplementary Table 1. Additionally, we manually searched reference lists of relevant articles, systematic reviews, and meta-analyses.

### 2.3 Inclusion criteria

(1) Study participants were adult men or women (≥18 years) with overweight or obesity, or additionally characterized by obesity-related metabolic risk phenotypes (such as metabolic syndrome (MetS), non-alcoholic fatty liver disease (NAFLD), type 2 diabetes mellitus (T2DM), or elevated cardiovascular risk); (2) The intervention group received purified hesperidin or hesperidin-containing citrus matrices/complexes as the intervention (type and duration unrestricted); (3) The control group received placebo or control intervention; (4) Primary outcome indicators included CRP or hsCRP, IL-6, TNF-α; oxidative stress indicators included TAC, MDA, and SOD; secondary outcome indicators included ICAM-1 and VCAM-1, with inclusion requiring reporting of at least one of these; (5) Study design was parallel or crossover RCTs. Studies were retained in the systematic review even if quantitative data for specific outcomes could not be used for pooled analysis.

### 2.4 Exclusion criteria

(1) Non-randomized or non-controlled studies; (2) Trial studies without eligible control groups; (3) Companion publications or duplicate publications of non-independent randomized cohorts; (4) Studies evaluating acute effects of hesperidin rather than sustained supplementation effects; (5) Studies that did not clearly report hesperidin dose or did not report target outcomes; (6) Non-original RCT evidence such as animal studies, *in vitro* studies, reviews, case reports, and conference abstracts.

### 2.5 Study selection and data extraction

Two reviewers (K. Z. and W. L.) independently screened titles and abstracts, assessed full-text eligibility, and extracted data using predefined standardized forms. Disagreements were resolved through consensus. Extracted data included first author name, publication year, country, study design, participant characteristics (number of participants per group, health status), intervention characteristics (dose, type, and duration), outcomes, adverse events, and funding sources.

To avoid unit-of-analysis errors, only one comparison and one effect size per study were retained in the primary pooled analysis for each outcome. When an outcome was reported at multiple post-intervention time points, the primary analysis selected only one time point: measurements at the end of the prespecified intervention period were prioritized; if not separately reported, the last available follow-up data were used. When a study included multiple intervention periods, the longest period was extracted; when a study included multiple intervention groups, data from the group most directly related to hesperidin-only intervention were extracted. When both endpoint values and change-from-baseline values were reported for the same comparison, the primary dataset prioritized the data basis that better preserved the trial's original analytical structure and variance information; for example, crossover trials prioritized paired or adjusted differences reported in the original text, while parallel trials prioritized endpoint or change values with complete information allowing standard error reconstruction. For each prespecified outcome indicator, meta-analysis was performed if two or more studies provided usable data, with results displayed in forest plots. If only one study reported an indicator, the study's results were presented directly without quantitative synthesis. All extraction results were cross-checked by two researchers, and disagreements were resolved through discussion with a third reviewer (Y.D. Y).

### 2.6 Risk of bias and evidence quality assessment

The Cochrane RoB 2 tool was used to assess risk of bias (24). Assessment covered five domains: randomization process, deviations from intended interventions, missing outcome data, measurement of the outcome, and selective reporting. Two reviewers (K. Z. and W. L.) independently assessed each domain, with risk levels classified as low risk, some concerns, or high risk. Results were cross-checked, and disagreements were resolved by a third reviewer (Y. D. Y.). The Grading of Recommendations Assessment, Development and Evaluation (GRADE) approach was used to assess evidence quality (25–27), with evidence quality classified into four levels: very low, low, moderate, and high.

### 2.7 Statistical analysis

Continuous outcomes were pooled as mean difference (MD) with 95% confidence interval (CI). For TAC, because studies used different assay methods, we used standardized mean difference (SMD; Hedges' *g*). For crossover trials, we used paired effect estimates or reported adjusted summaries when available. When paired variances were not reported, we conservatively treated crossover studies as parallel-group comparisons (28). This approach avoids overestimating precision when within-participant correlations are unavailable, but it may reduce statistical efficiency and yield wider CIs than analyses using the correct paired variance. We pooled endpoint and change-from-baseline data in the primary analysis; reporting-basis-related checks were retained as supplementary sensitivity analyses rather than core main-text sensitivity analyses. When standard deviations were not reported, we used established methods to derive missing variance terms from standard errors, CIs, or interquartile ranges (29–32). For studies reporting medians and interquartile ranges, Wan/Luo conversion methods were used to estimate means and standard deviations (31, 32). Between-study heterogeneity was assessed using Cochran's *Q* test and *I*^2^ statistic, with *I*^2^ > 50% or *P* < 0.10 considered to indicate significant heterogeneity, prompting use of random-effects models for pooling effect sizes; otherwise, fixed-effect models were used. For outcomes with very few studies, incomplete crossover design information, or mixed data bases, result interpretation considered study number, effect direction, and data structure together. When study number and distribution permitted, we conducted exploratory subgroup analyses for primary inflammatory indicators, with grouping factors including study type, intervention type, duration, dose, health status, presence of lifestyle co-interventions, and risk of bias; if any subgroup stratum had too few studies, only descriptive interpretation was provided. Dose and duration response analyses for CRP/hsCRP, IL-6, and TNF-α were conducted as exploratory meta-regression extensions and interpreted as hypothesis-generating results (33). Leave-one-out sensitivity analysis was used to assess result stability. For outcomes with no fewer than 10 included studies, potential publication bias was assessed through funnel plots, Begg's test, and Egger's test; if publication bias was suggested, the trim-and-fill method was used to explore potential impact (34–36). All analyses were performed in R version 4.5.3 using metafor version 4.8–0. *P* < 0.05 was considered statistically significant.

## 3 Results

### 3.1 Study selection

**Figure 1:**
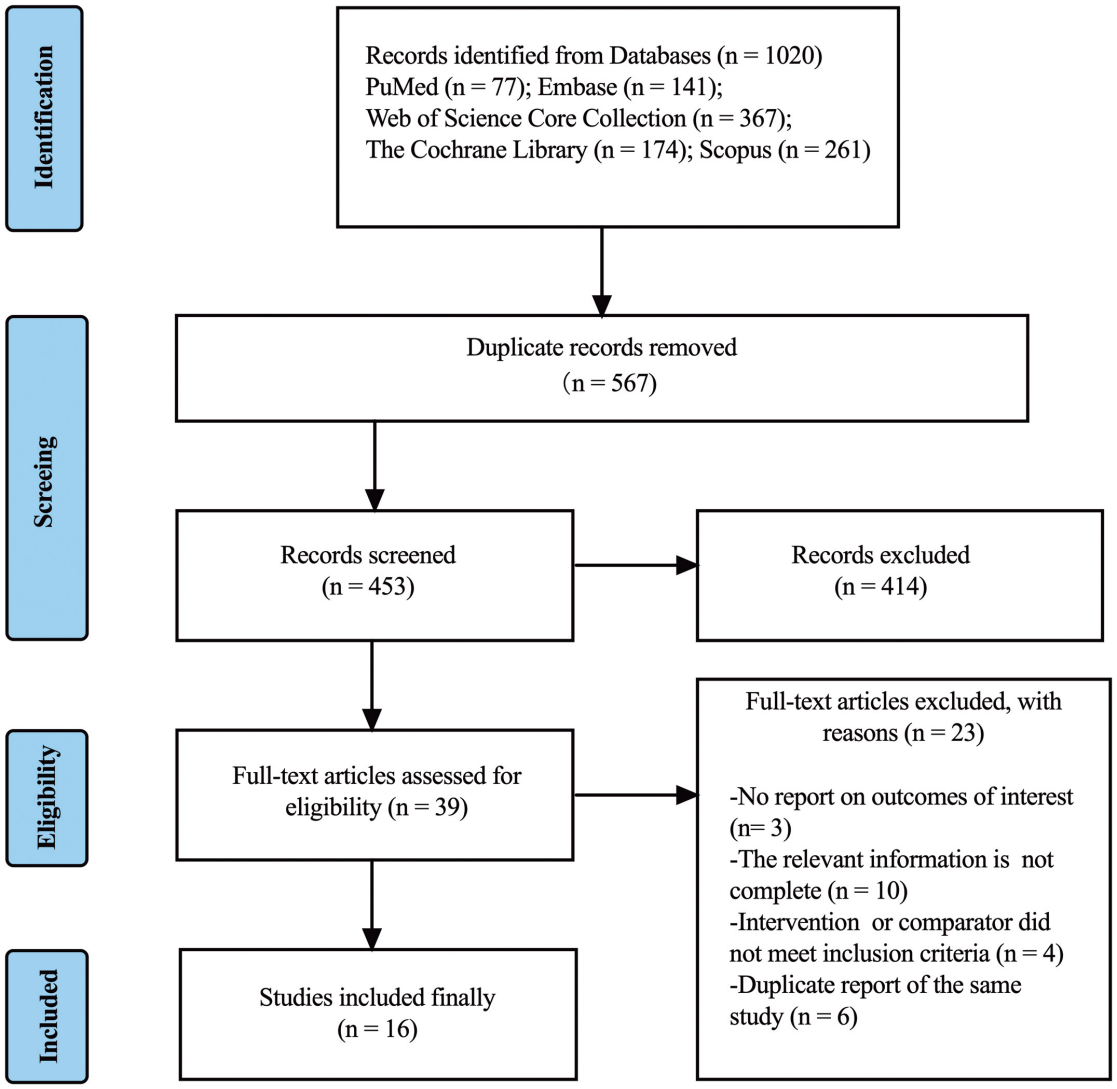
Flowchart of the literature screening process.

We retrieved a total of 1,020 records from 5 databases. After removing 567 duplicate records, we screened 453 records by title and abstract and excluded 414 obviously irrelevant records. Subsequently, 39 potentially relevant full-text reports were assessed for eligibility, and 23 full-text reports were excluded. Finally, 16 RCTs were included in the systematic review and meta-analysis. Reasons for full-text exclusion are listed in Supplementary Table 2, and the detailed flow diagram is shown in Figure 1.

### 3.2 Study characteristics

This review included 16 RCTs involving a total of 845 participants. Trials were conducted in the United Kingdom, France, Italy, the Netherlands, Brazil, Iran, and Spain. Overall, there were 10 parallel studies and 6 crossover studies; all crossover studies reported washout periods (3 days to 7 weeks), with 5 reporting no carryover effects. Participants included individuals who were healthy but overweight or obese, as well as those with obesity-related metabolic abnormalities such as T2DM, NAFLD, and MetS. Participant numbers ranged from 15 to 100, treatment duration ranged from 1 to 12 weeks, and daily hesperidin doses provided by the interventions ranged from 61.7 mg to 1,000 mg. Seven studies used purified hesperidin preparations, whereas nine used hesperidin-containing citrus matrices or complexes, including orange juice and whole oranges. Seven studies combined the intervention with lifestyle (diet and exercise) modifications. Only one study reported adverse events (one participant developed a rash, which resolved after discontinuation). Most trials reported no conflicts of interest, but funding sources varied and included public, university, and industry- or citrus-sector-related support. Detailed study characteristics are shown in Table 1.

**Table 1:** Study characteristics of the included trials.

Study	Country and population	Design and analyzed sample	Intervention/ comparator	Duration (weeks)	Main inflammatory or oxidative outcomes contributed within the review	Funding
(68)	Italy; nondiabetic adults with increased cardiovascular risk	Single-blind placebo-controlled crossover; 19 completers	500 mL/d red OJ (hesperidin: 159.5 mg/d) vs. matched placebo drink	1 + 3 d washout period	CRP/hsCRP, IL-6, TNF-α	No funding reported
(69)	Iran; adults with NAFLD	Double-blind parallel trial; analyzed 25/24	Hesperidin capsules 1,000 mg/d plus lifestyle advice vs. placebo capsules plus the same lifestyle advice	12	CRP/hsCRP, TNF-α	NR
(70)	France; mild hypercholesterolemic men with cardiovascular risk factors	Single-blind crossover trial; 25 completers	600 mL/d blood OJ (hesperidin: 212 mg/d) vs. control beverage	4 + 5 wk washout period	CRP/hsCRP, ICAM-1, VCAM-1 (supplementary quantitative analyses only)	French National Research Agency (ANR), PNRA
(71)	Iran; adults with T2DM	Double-blind parallel trial; analyzed 31/29	500 mg/d hesperidin capsule vs. placebo capsule (starch)	6	CRP/hsCRP, IL-6, TNF-α, TAC	Nutrition and Metabolic Disease Research Center of Ahvaz Jundishapur University of Medical Sciences, Grant NRC-9411
(72)	United Kingdom; healthy adults with overweight or obesity	Single-blind crossover trial; 15 completers overall, with the hsCRP subset retained for quantitative use	400 mL/d blood OJ (hesperidin: 320.8 mg/d) vs. control drink (hesperidin: 25.2 mg/d)	2 + 1 wk washout period	CRP/hsCRP	China Scholarship Council-University of Leeds Scholarship
(73)	France; healthy overweight men	Three-period crossover trial; 23 completers	500 mL/d control drink plus 292 mg/d hesperidin vs. 500 mL/d control drink plus placebo (starch)	4 + 3 wk washout period	CRP/hsCRP, IL-6; supplementary ICAM-1 and VCAM-1 contrasts	Florida Department of Citrus
(74)	Spain; adults with obesity	Double-blind parallel trial; analyzed 22/20 in the responder-only per-protocol set	200 mL/d OJ (hesperidin: 143.8 mg/d) with a reduced-calorie diet vs. 200 mL/d placebo juice (hesperidin: 61.7 mg/d) with a reduced-calorie diet	6	CRP/hsCRP	NR
(75)	Italy; adults with metabolic dysfunction-associated steatotic liver disease (MASLD) and overweight	Parallel RCT; analyzed 31/31	400 g/d whole oranges (hesperidin: 202.3 mg/d) vs. non-citrus fruits 400 g/d	4	CRP/hsCRP	Italian Ministry of Health Ricerca Corrente 2024
(76)	Brazil; adults with MetS	Parallel-group RCT; analyzed 36/36	500 mL/d OJ (hesperidin: 121.6 mg/d) plus balanced diet vs. balanced diet	12	CRP/hsCRP, TAC	CitrusBR, CAPES, Citrosuco S.A.
(77)	Spain; overweight or obese nonsmoking adults	Double-blind crossover trial; 100 completers in the nonsmoking analysis set	500 mL/d high-polyphenol OJ (hesperidin: 582.5 mg/d) vs. 500 mL/d regular-polyphenol OJ (hesperidin: 237.5 mg/d)	12 + 7 wk washout period	SOD (supplementary quantitative analyses only)	Coca-Cola Europe, research contract 3345
(78)	Brazil; adults with obesity	Parallel-group RCT; analyzed 39/39	500 mL/d of OJ (hesperidin: 162 mg/d) with a reduced-calorie diet vs. reduced-calorie diet	12	CRP/hsCRP, TAC, MDA	Citrosuco S.A., PADC/FCFAr, CAPES
(79)	Italy; adults with MetS	Double-blind placebo-controlled crossover trial; 24 completers	500 mg/d hesperidin capsule vs. placebo capsule (cellulose)	3 + 3 d washout period	CRP/hsCRP; supplementary ICAM-1 and VCAM-1	NR
(80)	Netherlands; healthy overweight adults	Double-blind parallel trial; analyzed 33/32	450 mg/d hesperidin 2S capsule vs. placebo capsule (cellulose)	6	ICAM-1, VCAM-1 (supplementary quantitative analyses only)	BioActor BV
(81)	United Kingdom; overweight men with elevated fasting cholesterol	Single-blind parallel trial; analyzed 18/18	250 mL/d OJ (hesperidin: 135.4 mg/d) vs. 250 mL/d control drink (hesperidin: 0)	12	CRP/hsCRP, IL-6, TNF-α	Florida Department of Citrus
(82)	Iran; adults with MetS	Double-blind parallel trial; analyzed 25/24	1,000 mg/d hesperidin capsule plus lifestyle modification program vs. placebo capsule (starch) plus lifestyle modification program	12	CRP/hsCRP, TNF-α	Student Research Committee, Shahid Beheshti University of Medical Sciences
(83)	Iran; adults with NAFLD	Four-arm randomized trial; analyzed 22/21 for the hesperidin-only vs control comparison	1,000 mg/d hesperidin capsule plus lifestyle modification program vs. lifestyle modification program	12	CRP/hsCRP, TNF-α	NR

CRP, c-reactive protein; d, day; hsCRP, high-sensitivity C-reactive protein; IL-6, interleukin-6; ICAM-1, intercellular adhesion molecule 1; MASLD, metabolic dysfunction-associated steatotic liver disease; MetS, metabolic syndrome; MDA, malondialdehyde; NAFLD, non-alcoholic fatty liver disease; NR, not reported; OJ, orange juice; RCT, randomized controlled trial; SOD, superoxide dismutase; T2DM, type 2 diabetes mellitus; TAC, total antioxidant capacity; TNF-α, tumor necrosis factor alpha; VCAM-1, vascular cell adhesion protein 1; wk, week.

### 3.3 Risk of bias assessment

**Figure 2:**
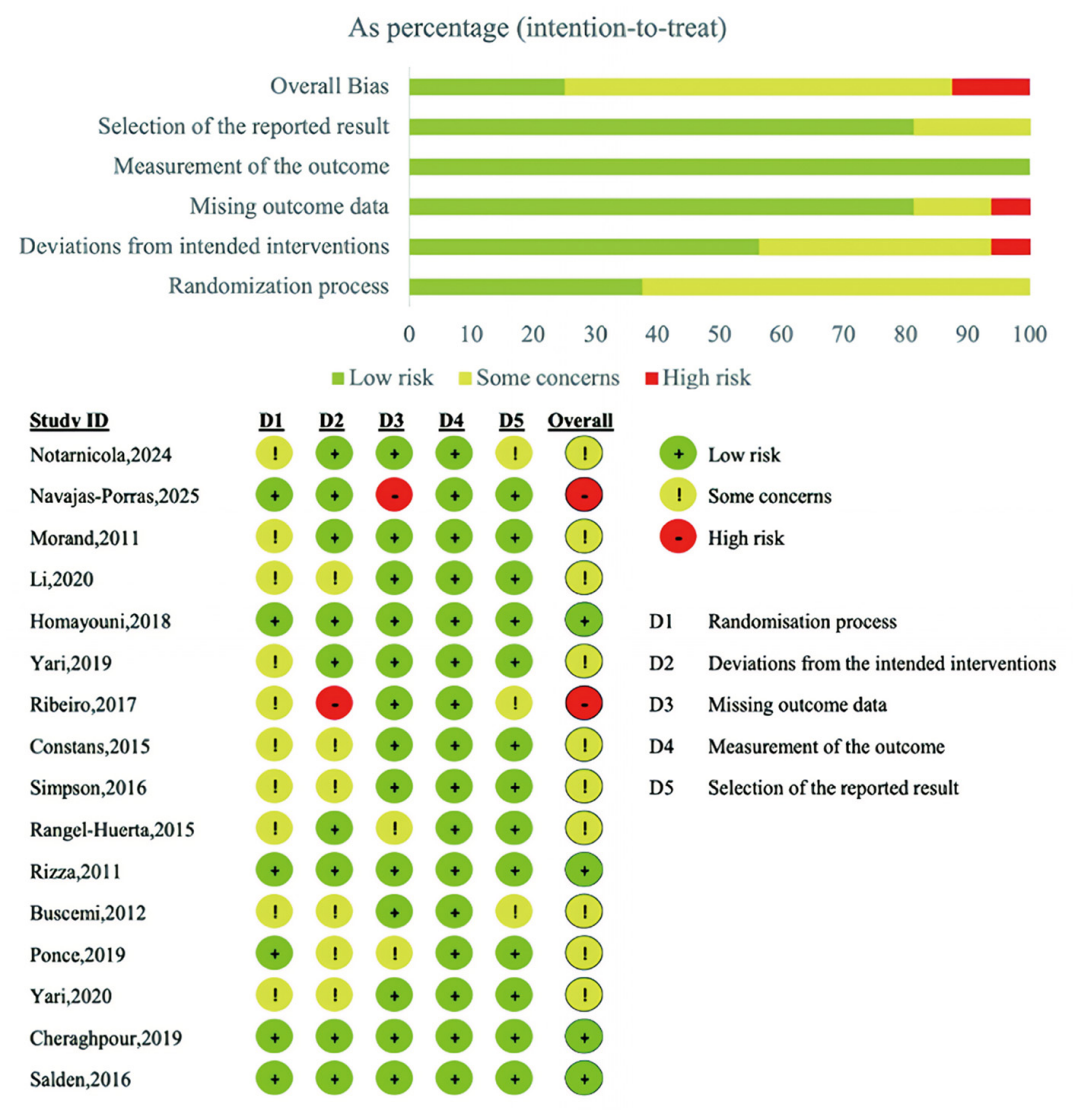
Visualization and traffic light plots of the risk of bias assessment using RoB 2.

We assessed the risk of bias of the 16 included studies using the Cochrane RoB 2 tool (24). Overall, 25% of studies were rated as low risk of bias, 62.5% had some concerns, and 12.5% had high risk of bias. Across all studies, “measurement of the outcome” was considered low risk. “Randomization process” was the most common source of concern, with 10 studies rated as having some concerns due to insufficient description of random sequence generation, allocation concealment, or allocation implementation details. Regarding “deviations from intended interventions,” 6 studies had some concerns, mainly related to single-blind, open-label designs, or food matrix interventions that were difficult to fully blind; 1 study was rated as high risk due to lack of blinding, completer analysis, and absence of intention-to-treat analysis. Regarding “missing outcome data,” 2 studies had some concerns, and 1 study was rated as high risk due to substantial post-randomization exclusions and responder-only analysis. For “selective reporting,” 13 studies were low risk, and 3 studies had some concerns due to lack of verifiable prespecified analysis plans or registration information. The RoB 2 visualization and traffic light plot for risk of bias assessment are shown in Figure 2.

### 3.4 GRADE evidence quality assessment

The GRADE approach was used to assess certainty of evidence for the primary and supportive outcomes listed in Table 2. CRP/hsCRP was the only outcome achieving moderate certainty; IL-6, TNF-α, TAC, MDA, SOD, ICAM-1, and VCAM-1 all had very low certainty of evidence, primarily limited by risk of bias, between-study heterogeneity, imprecision, assay method differences, indirectness, or single-study evidence.

**Table 2:** GRADE evidence profile for the main outcomes.

Outcomes	Risk of bias	Inconsistency	Indirectness	Imprecision	Publication bias	Quality of evidence
CRP/hsCRP	Serious	Not serious	Not serious	Not serious	Not serious	⊕⊕⊕° Moderate
IL-6	Serious	Serious	Not serious	Serious	Not assessable	⊕°°° Very low
TNF-α	Serious	Serious	Not serious	Serious	Not assessable	⊕°°° Very low
TAC	Serious	Serious	Serious	Serious	Not assessable	⊕°°° Very low
MDA	Serious	Not applicable	Not serious	Very serious	Not assessable	⊕°°° Very low
ICAM-1	Serious	Serious	Serious	Very serious	Not assessable	⊕°°° Very low
VCAM-1	Serious	Serious	Serious	Very serious	Not assessable	⊕°°° Very low
SOD	Serious	Not applicable	Very serious	Very serious	Not assessable	⊕°°° Very low

### 3.5 Meta-analysis results

#### 3.5.1 Effects of hesperidin-related interventions on CRP/hsCRP

**Figure 3:**
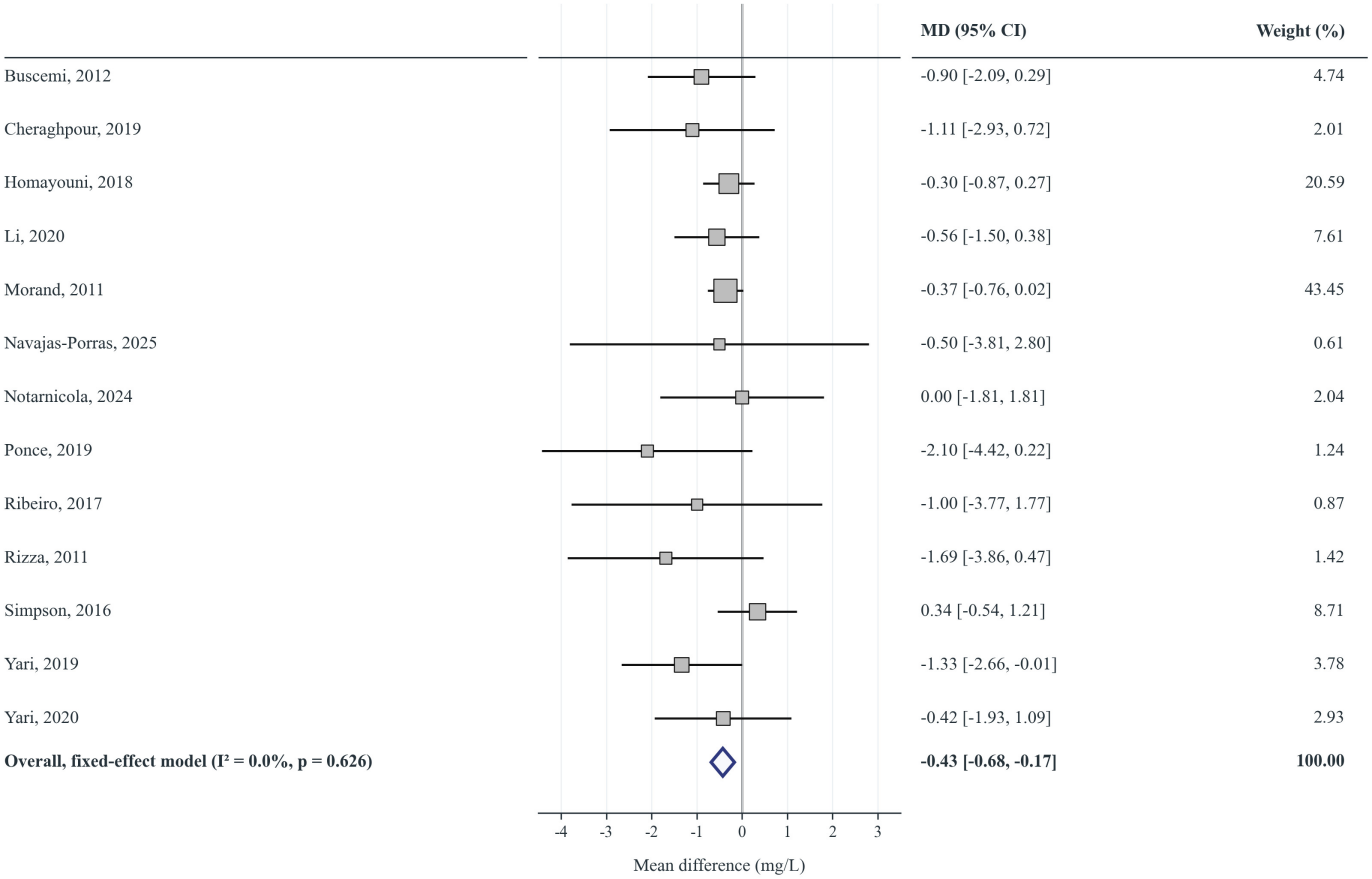
Forest plot of CRP/hsCRP.

Thirteen studies involving 643 participants reported the effects of hesperidin-related interventions on CRP/hsCRP. Fixed-effect model pooling showed that compared with placebo or control, hesperidin-related interventions significantly reduced CRP/hsCRP (MD: −0.43 mg/L, 95% CI: −0.68, −0.17, *P* = 0.001), with negligible heterogeneity (*I*^2^ = 0.0%, *P* = 0.626) (Figure 3). Exploratory subgroup analyses (Supplementary Table 3) showed that study type (*P* = 0.683), intervention type (*P* = 0.781), duration (*P* = 0.888), dose (*P* = 0.198), health status (*P* = 0.839), lifestyle co-intervention (*P* = 0.077), and risk of bias (*P* = 0.917) did not show clear effect modification. The subgroup with lifestyle co-interventions showed numerically greater reduction, but the formal interaction did not reach statistical significance.

#### 3.5.2 Effects of hesperidin-related interventions on TNF-α

**Figure 4:**
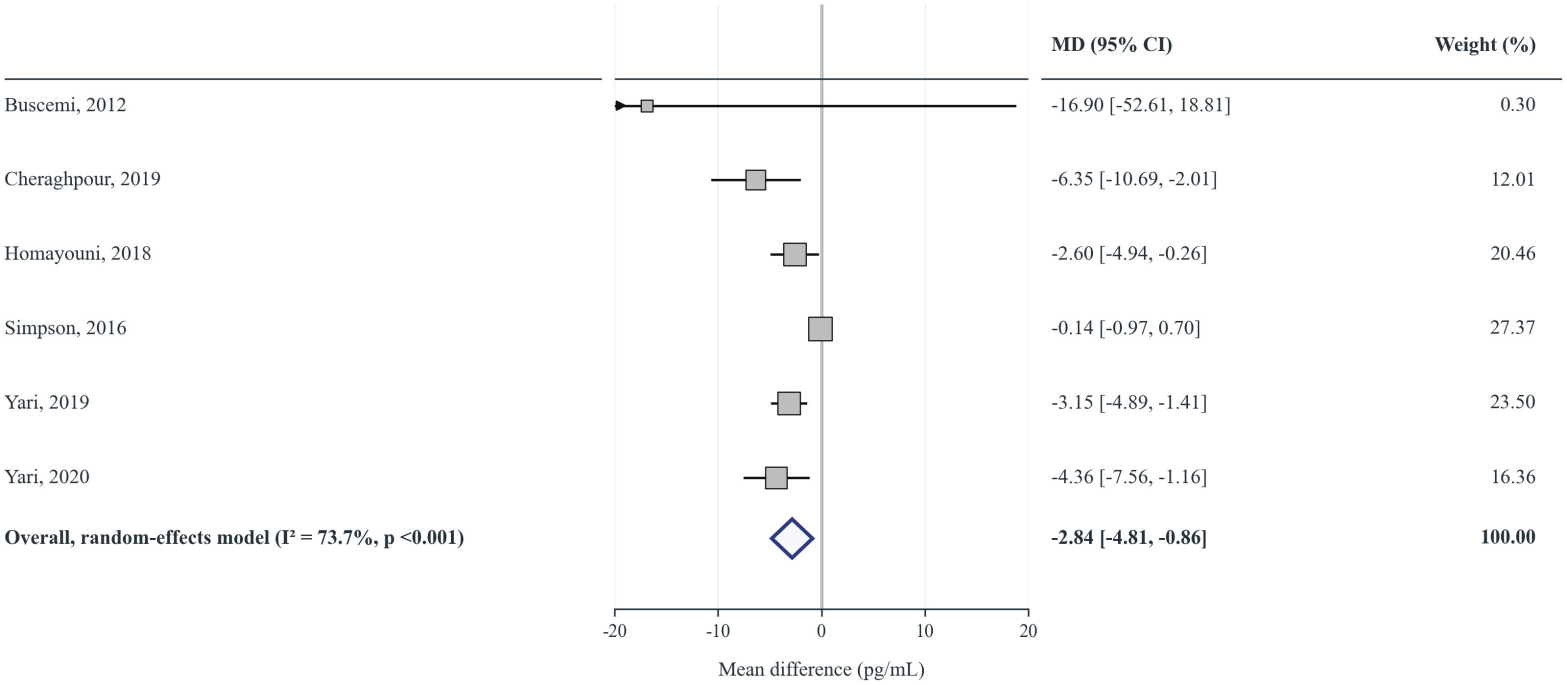
Forest plot of TNF-α.

This analysis included 6 studies (275 participants), and results showed that hesperidin-related interventions significantly reduced TNF-α (MD: −2.84 pg/mL, 95% CI: −4.81, −0.86, *P* = 0.005), but between-study heterogeneity was observed (*I*^2^ = 73.7%, *P* < 0.001) (Figure 4). Exploratory subgroup analyses showed numerical differences across intervention type (*P* < 0.001), dose stratum (*P* = 0.039), and lifestyle co-intervention (*P* = 0.039); purified hesperidin, the >500 mg/day stratum, and studies with lifestyle co-interventions showed numerically larger reductions. However, these patterns were based on few studies with overlapping characteristics and high residual heterogeneity, and should be interpreted as exploratory signals rather than evidence of formulation, dose-stratum, or co-intervention superiority (Supplementary Table 3).

#### 3.5.3 Effects of hesperidin-related interventions on IL-6

**Figure 5:**
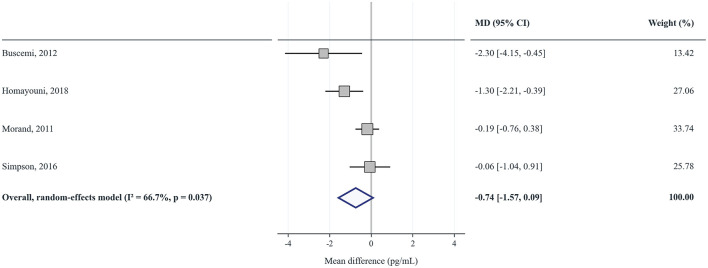
Forest plot of IL-6.

Four studies (180 participants) were included in the IL-6 analysis. Random-effects model pooling showed that hesperidin-related interventions had no significant effect on IL-6 (MD: −0.74 pg/mL, 95% CI: −1.57, 0.09, *P* = 0.081), with between-study heterogeneity present (*I*^2^ = 66.7%, *P* = 0.037) (Figure 5). Subgroup analyses showed that study type (*P* = 0.814) and intervention type (*P* = 0.835) did not show interaction signals; other grouping factors had too few studies per stratum and were only descriptively presented, not used to judge stable effect modification (Supplementary Table 3).

#### 3.5.4 Effects of hesperidin-related interventions on oxidative stress indicators

Three studies using different assay methods were included in the TAC analysis, and results showed that hesperidin-related interventions had no significant effect on TAC (SMD: −0.19, 95% CI: −0.78, 0.40, *P* = 0.530), with between-study heterogeneity present (*I*^2^ = 77.6%, *P* = 0.010) (Supplementary Figure 1A). Ribeiro 2017 was the only study reporting MDA, which compared 12 weeks of 500 mL/d orange juice plus reduced-calorie diet vs. reduced-calorie diet alone in Brazilian adults with obesity. MDA decreased from baseline in both groups (orange juice group 1.5 ± 0.9 to 0.8 ± 0.4 mM; control group 1.7 ± 0.8 to 0.9 ± 0.5 mM), with both groups showing approximately 47% reduction, but no clear between-group difference was suggested. The single-study between-group estimate in this review was MD: −0.10 mM (95% CI: −1.36, 1.16), with small and imprecise difference (Supplementary Figure 1B). Only Rangel-Huerta 2015 reported SOD results, which was an active-control crossover comparison of high-polyphenol orange juice (containing hesperidin 582.5 mg/d) vs. regular-polyphenol orange juice (containing hesperidin 237 mg/d); the displayable between-group estimate was MD: 2.00 U/mg Hb (95% CI: −2.71, 6.71) (Supplementary Figure 1C). Due to lack of reusable paired crossover effect estimates and non-significant intervention effects and interactions reported in the original text, this review did not fit a pooled SOD model and cannot support consistent improvement in SOD activity by hesperidin-related interventions based on this.

#### 3.5.5 Effects of hesperidin-related interventions on endothelial function markers

Exploratory pooling of ICAM-1 and VCAM-1 included only 4 independent studies. Fixed-effect model showed that hesperidin-related interventions had no significant effect on ICAM-1 (MD: −4.06 ng/mL, 95% CI: −13.59, 5.47, *P* = 0.404), with negligible heterogeneity (*I*^2^ = 0.0%, *P* = 0.811) (Supplementary Figure 2A). VCAM-1 pooling results suggested significant reduction (MD: −27.16 ng/mL, 95% CI: −50.13, −4.19, *P* = 0.020), with no obvious between-study heterogeneity (*I*^2^ = 0.0%, *P* = 0.442) (Supplementary Figure 2B). However, this analysis mixed crossover-adjusted effects, conservative endpoint approximations, and special formulation studies, with small study numbers and very low certainty of evidence; therefore, it should be interpreted as an exploratory supplementary signal rather than definitive endothelial function benefit.

#### 3.5.6 Exploratory dose and duration response analyses

We conducted exploratory dose- and duration-response analyses for primary inflammatory biomarkers. CRP/hsCRP showed no clear dose or duration signals: in dose models including all intervention forms, meta-regression coefficient *P* values were 0.975 (linear term) and 0.737 (quadratic term); duration model *P* values were 0.305 and 0.317; purified hesperidin subset dose model *P* values were 0.791 and 0.643 (Supplementary Figure 3). IL-6 showed no clear dose or duration signals (meta-regression coefficient P values were 0.730 and 0.299, respectively) (Supplementary Figure 4). Exploratory dose models for TNF-α showed a statistical pattern compatible with larger reductions at higher nominal doses (meta-regression coefficient *P* < 0.001), but this finding is highly vulnerable to confounding by formulation and co-intervention; duration showed no association (*P* = 0.955) (Supplementary Figure 5). Additional crossover contrasts from Morand 2011 were displayed as separate supplements only (Supplementary Figure 6). These results are affected by small study numbers and limited dose comparability across different intervention forms, and should be viewed as exploratory and hypothesis-generating findings. Complete dose- and duration-response results are shown in Supplementary Table 4.

### 3.6 Sensitivity analyses

Sensitivity analysis results showed that the effect size of hesperidin-related interventions on CRP/hsCRP remained robust even when studies were removed one by one; after excluding high risk of bias studies, results remained similar (MD: −0.42 mg/L, 95% CI: −0.68, −0.16). IL-6 remained uncertain in leave-one-out analysis, with CIs crossing the null line after each removal. TNF-α maintained a favorable direction in leave-one-out analysis, but heterogeneity remained high except when Simpson 2016 was removed. TAC was sensitive to study composition; after removing high risk of bias Ribeiro 2017, pooled results from the remaining two studies showed lower TAC in the intervention group (SMD: −0.49, 95% CI: −0.85, −0.14), and therefore should not be interpreted as antioxidant benefit; this directional change further weakens the stability of evidence for TAC improvement. Complete sensitivity analysis summary is shown in Supplementary Table 5. Supplementary reporting-basis and data-handling sensitivity figures are shown in Supplementary Figures 7–12.

### 3.7 Publication bias

For the CRP/hsCRP outcome, the funnel plot appeared roughly symmetric visually, with Begg's test *P* = 0.129 and Egger's test *P* = 0.114, neither suggesting significant publication bias (Supplementary Figure 13). Due to small numbers of included studies for other outcome indicators, reliable publication bias assessment could not be performed.

## 4 Discussion

This systematic review and meta-analysis found that hesperidin-related interventions were associated with a modest reduction in CRP/hsCRP in individuals with overweight or obesity. CRP/hsCRP was the only outcome simultaneously exhibiting favorable pooled direction, negligible heterogeneity, and moderate-certainty evidence. TNF-α and VCAM-1 also favored intervention in pooled analyses, but these findings were less certain because they were based on fewer studies, greater data-structure limitations, high heterogeneity for TNF-α, and very low certainty of evidence. IL-6, TAC, MDA, ICAM-1, and SOD did not support clear or consistent improvement.

The population focus of this review is clinically relevant. Overweight and obesity are not merely anthropometric states but represent chronic inflammatory phenotypes associated with metabolic and vascular risk (37–39). Modest but consistent CRP reduction may be more informative than isolated changes in the cytokine pool, because CRP integrates inflammatory signaling and is part of the residual inflammatory risk framework in cardiometabolic prevention (5–7). The included trials recruited individuals with overweight or obesity, with some populations further characterized by MetS, NAFLD, or T2DM. Therefore, this meta-analysis maps to a clinically heterogeneous but highly relevant obesity risk spectrum.

The 0.43 mg/L reduction in CRP/hsCRP should be interpreted within the residual inflammatory risk framework but not as evidence of direct clinical benefit. The JUPITER trial showed that in populations with low-density lipoprotein cholesterol (LDL-C) not elevated but hsCRP ≥2.0 mg/L, rosuvastatin reduced hsCRP and major cardiovascular events (40); the CANTOS trial further demonstrated that targeted anti-inflammatory therapy independent of lipid lowering could reduce recurrent cardiovascular events (41). Epidemiological pooled studies also support a continuous association between CRP and risk of coronary heart disease and vascular events (42, 43), and the American Heart Association/Centers for Disease Control and Prevention (AHA/CDC) statement uses hsCRP < 1, 1–3, and >3 mg/L for low, moderate, and high risk stratification, respectively (44). In this context, the observed CRP/hsCRP reduction is best interpreted as a modest biomarker-level signal that may be relevant to nutrition-based adjunctive strategies in individuals with elevated baseline CRP, including trial populations in this review with baseline CRP approximately 3–4 mg/L. However, clinical implications require confirmation in longer-term trials with clinical endpoints.

IL-6 did not reach statistical significance. IL-6 is positioned at the upstream cytokine level, with circulating levels influenced by sampling time, acute stress, and detection platforms; CRP is a more stable downstream acute-phase protein and integrative inflammatory readout (45–48). Therefore, different pooled results for IL-6 and CRP in the same intervention are not contradictory. Small study numbers and moderate heterogeneity further reduced the ability to capture mild changes in upstream cytokines. Other dietary polyphenol evidence also shows that upstream cytokine effects are often less stable than CRP; for example, IL-6 results for anthocyanin supplementation in obesity-related inflammatory profiles remain uncertain (49). Therefore, the negative IL-6 result in this study is more appropriately interpreted as failure to confirm stable effects under current conditions of small sample size, assay heterogeneity, and short-term intervention, rather than complete lack of anti-inflammatory potential for hesperidin-related interventions.

TNF-α showed a statistically significant reduction, but the estimate was less stable than the CRP/hsCRP finding because it was based on fewer studies and substantial between-study heterogeneity. This pattern is biologically plausible given the role of TNF-α in obesity-related adipose tissue inflammation and metabolic dysfunction (50, 51), and is consistent with experimental evidence linking hesperidin and hesperetin to anti-inflammatory signaling. However, leave-one-out analysis indicated that heterogeneity was strongly influenced by individual studies, particularly Simpson 2016. Exploratory subgroup analyses showed numerical differences by intervention type, dose stratum, and lifestyle co-intervention, but these strata overlapped with formulation, dose standardization, population characteristics, and co-intervention background. Accordingly, the TNF-α result should be interpreted as a supportive inflammatory biomarker signal rather than evidence that any specific matrix, dose stratum, or co-intervention strategy is superior. This cautious interpretation is also consistent with the known sensitivity of TNF-α measurement to sample handling and detection platforms (45) and with mixed TNF-α findings reported for other dietary polyphenols (49, 52).

Mechanistic and bioavailability evidence helps contextualize the pooled findings. Hesperidin and hesperetin have been linked to inflammatory, oxidative stress, and endothelial pathways, while human studies indicate that flavanone metabolite exposure may vary across orange juice, whole-fruit, standard supplement, micronized, and 2S-enriched formulations (13–15, 53–55).ff These considerations support the biological plausibility of the CRP/hsCRP signal and help explain why nominal dose did not map clearly onto response across mixed purified and citrus-matrix interventions. They also support interpreting capsule-vs.-citrus contrasts as exploratory formulation hypotheses rather than fixed biological hierarchies.

The remaining inflammatory and oxidative stress outcomes showed less consistent patterns than CRP/hsCRP. A recent adult inflammation meta-analysis similarly found no overall benefit for IL-6 despite reductions in CRP/hsCRP and TNF-α, and suggested that IL-6 improvement may be more evident in diseased adults than in healthy adults (18). In the present dataset, supportive biomarker pools were smaller, more heterogeneous, and more sensitive to assay selection, intervention duration, population mix, and reporting basis than the CRP/hsCRP pool. CRP, as a downstream acute-phase marker with clearer clinical anchoring, is more stable across trials than upstream cytokines or sparse oxidative stress assays (4–6, 56).

The uncertainty in oxidative stress readouts also warrants separate emphasis. TAC is a context-dependent composite construct, and MDA assays have long-standing analytical reliability concerns (57, 58). This interpretation is consistent with broader trial analysis literature: endpoint values, change values, and analysis of covariance (ANCOVA)-based estimates differ in efficiency and may lead to different meta-analytic conclusions when evidence is sparse and reporting is inconsistent (59–61). Therefore, current results for TAC, MDA, and SOD are more suitable as a basis for future trial design rather than as definitive evidence that hesperidin-related interventions improve oxidative stress.

For clinicians, these findings do not yet support recommendations for a specific hesperidin formulation, citrus matrix, or dose, but they identify a modest CRP/hsCRP biomarker signal that may be relevant to nutrition-based adjunctive strategies in metabolically at-risk adults. For nutrition researchers, the findings highlight the need for trials that separate purified hesperidin effects from citrus-matrix effects, standardize inflammatory and oxidative stress assays, report paired variance for crossover designs, and clearly document co-interventions.

This review has several methodological strengths. First, it focused on at-risk populations with overweight/obesity while retaining both purified hesperidin and hesperidin-containing citrus matrices, which improves translational relevance. Second, the outcome hierarchy combined CRP/hsCRP as the primary anchor with upstream cytokines, oxidative stress readouts, and endothelial markers, providing a broader view of obesity-related inflammatory and redox biology. Third, the statistical approach was transparent, including differentiated handling of crossover and parallel designs, sensitivity checks for reporting basis, leave-one-out analyses, and exploratory subgroup analyses. Fourth, RoB 2 and GRADE provided an outcome-specific evidence strength hierarchy for interpretation.

Several limitations should be noted. First, structural heterogeneity remains the main limitation of the evidence base rather than sample size alone. Included trials differed in participant phenotype, purified vs. citrus-matrix interventions, control conditions, co-interventions, assay methods, reporting formats, and crossover-design variance reporting; these differences constrain attribution to hesperidin itself and limit direct comparisons across matrices, formulations, and dose strata.

Second, crossover trials often lacked paired variance or covariance information, so conservative parallel-group approximations were necessary and may have reduced statistical efficiency and widened CIs.

Third, intervention durations were short (1–12 weeks), and longer trials with clinical or sustained biomarker endpoints are needed to clarify the implications of the observed CRP/hsCRP change.

Fourth, biomarker measurement and reporting differed across studies, including assay platforms, sample handling, endpoint vs. change-score reporting, and exposure quantification; these differences limit dose-response interpretation and matrix-specific recommendations.

Future trials should move in more targeted directions. Populations with clearly defined inflammatory burden at baseline, standardized measurements of CRP/hsCRP and cytokines, and longer follow-up will be particularly important. For oxidative stress outcomes, assay harmonization will be as important as sample size, because TAC and MDA are not single interchangeable laboratory constructs (57, 58). Head-to-head comparisons of purified hesperidin, citrus beverages, and whole-fruit approaches will help distinguish hesperidin-specific signals from broader citrus-matrix signals, and clearer quantification of hesperidin or hesperetin exposure will improve dose interpretation (15, 54–56, 62–67). Better reporting of crossover covariance or paired variance, and clearer separation between lifestyle co-interventions and the test intervention itself, will also make future trials easier to synthesize and more clinically interpretable.

## 5 Conclusion

In summary, hesperidin-related interventions were associated with a modest reduction in CRP/hsCRP in adults with overweight or obesity. Evidence for TNF-α, VCAM-1, IL-6, oxidative stress, and endothelial markers remains less certain. Because the included trials differed in population, formulation, citrus matrix, co-interventions, assay methods, and crossover reporting, current evidence does not support specific recommendations by dose or matrix. Future randomized trials with longer follow-up, standardized biomarker measurements, clearer exposure quantification, and more complete crossover-design variance reporting are needed.
