# Supplementary material for: Effects of hesperidin supplementation on inflammation and oxidative stress in overweight or obese individuals: a systematic review and meta-analysis of randomized controlled trials
Source: Front Nutr. 2026 Jun 26;13:1871474. doi: 10.3389/fnut.2026.1871474 (PMC13350032; doi:10.3389/fnut.2026.1871474)
Supplement: Supplementary file 1 [file Supplementary_File_1.docx]

**
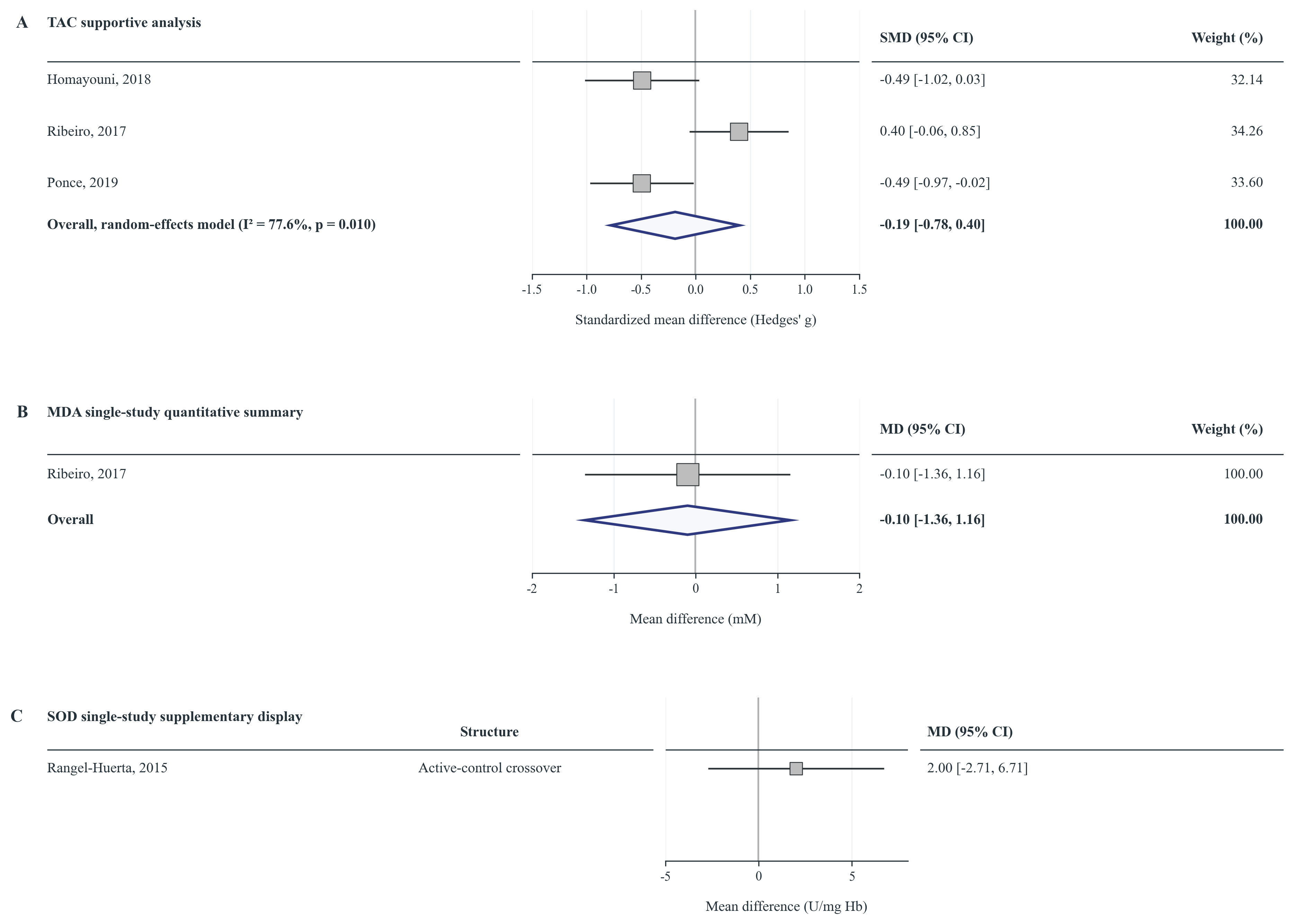
Supplementary Material**

**1.Supplementary Figures**

**Supplementary Figure 1. Supplementary quantitative summaries of TAC, MDA, and SOD**

Panel A shows TAC pooled as a standardized mean difference (Hedges’ g) because assay methods differed across studies. Panel B shows the single-study MDA group-difference summary from Ribeiro 2017 and was not pooled. Panel C shows the single-study SOD active-control crossover display from Rangel-Huerta 2015 and was not pooled. For MDA and SOD, effect estimates are mean differences in the native units shown on the x axes.

**
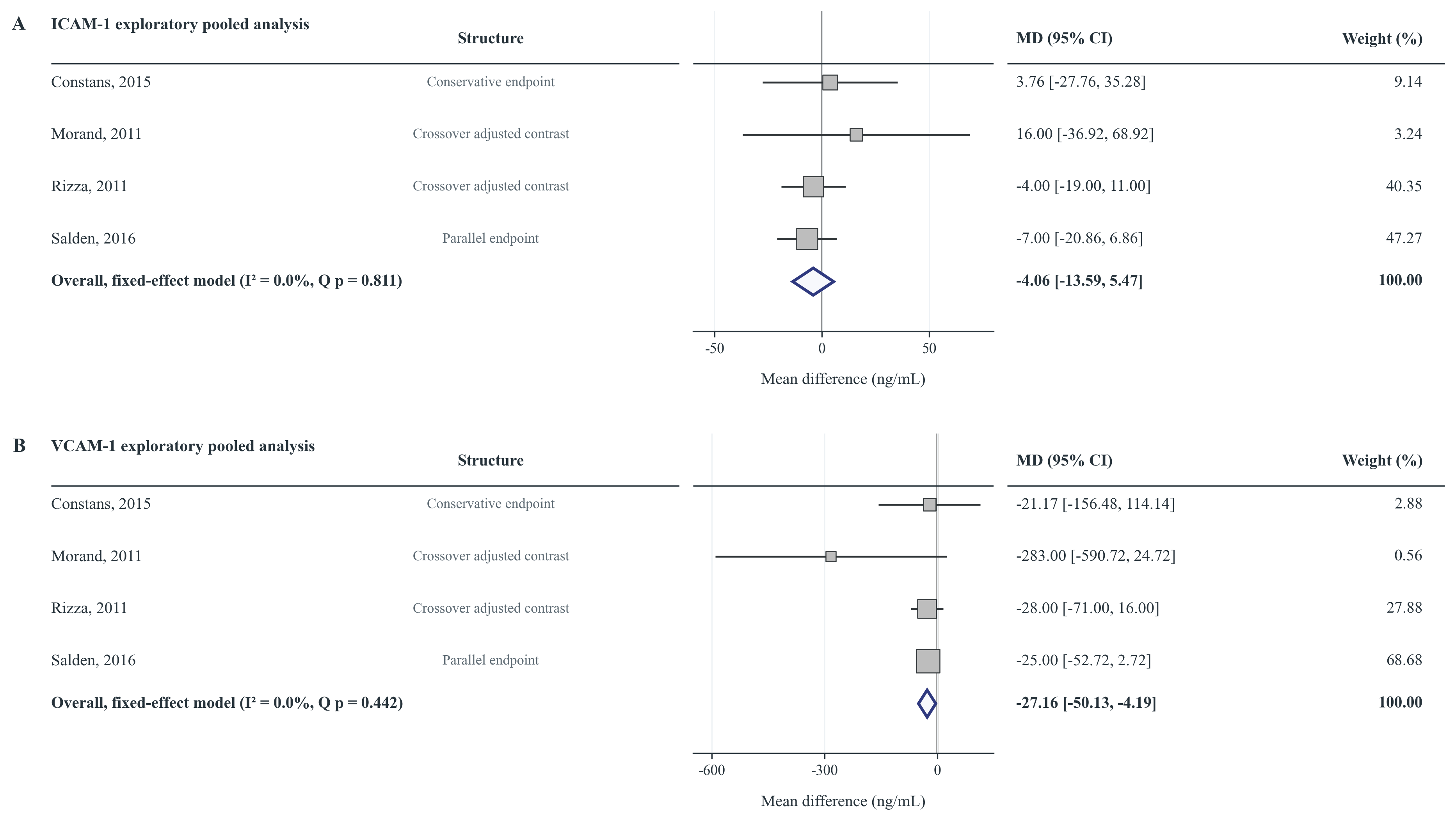
Supplementary Figure 2.** Exploratory pooled displays of endothelial adhesion markers. Panel A shows ICAM-1, and Panel B shows VCAM-1. Both analyses include 4 unique studies. Effect estimates are mean differences in ng/mL.


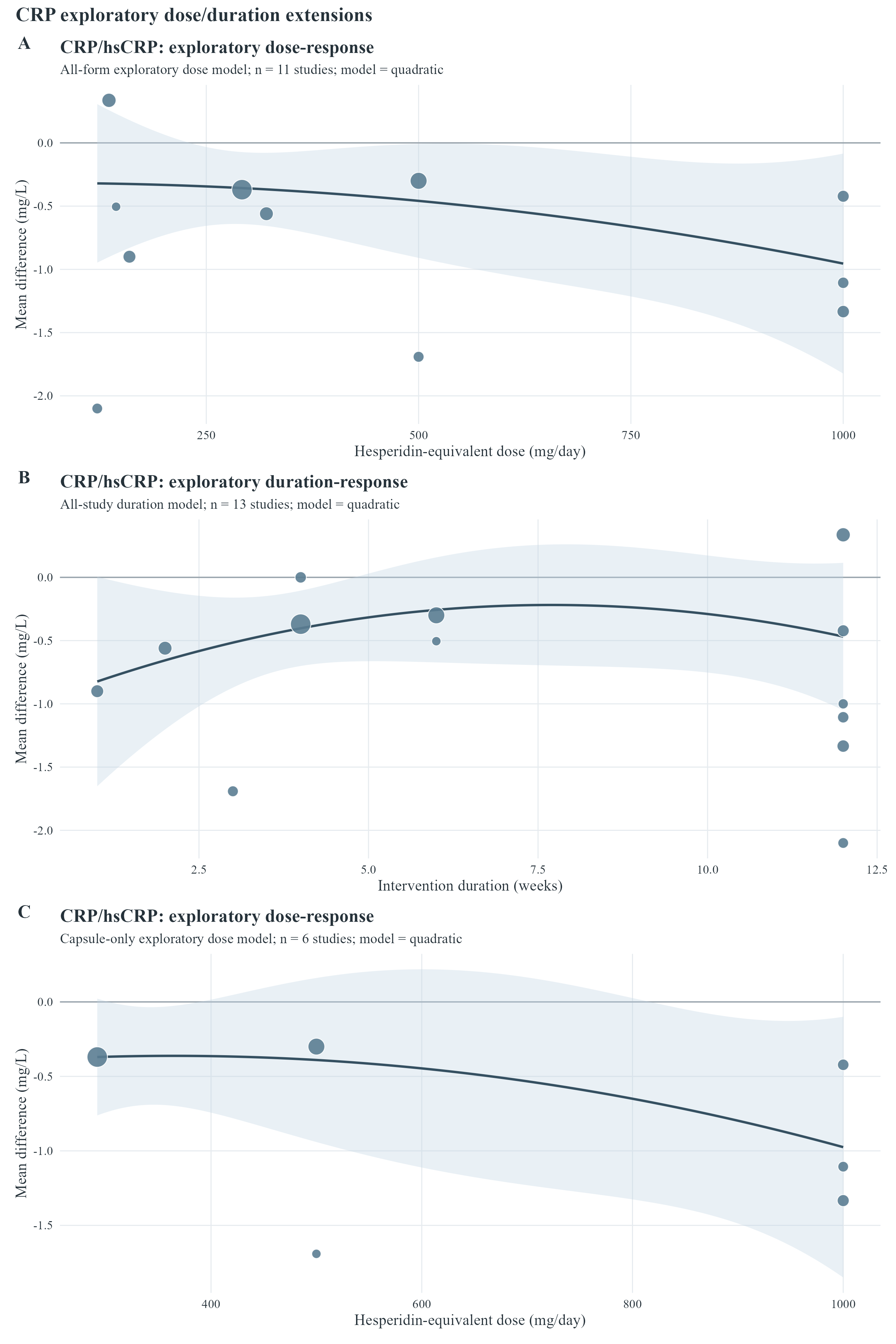


**Supplementary Figure 3**. Exploratory dose- and duration-response analyses of CRP/hsCRP. Panel A shows the all-form dose-response model, Panel B shows the duration-response model, and Panel C shows the capsule-only dose-response model. Curves represent exploratory meta-regression fits with 95% confidence bands; point size reflects study weight.


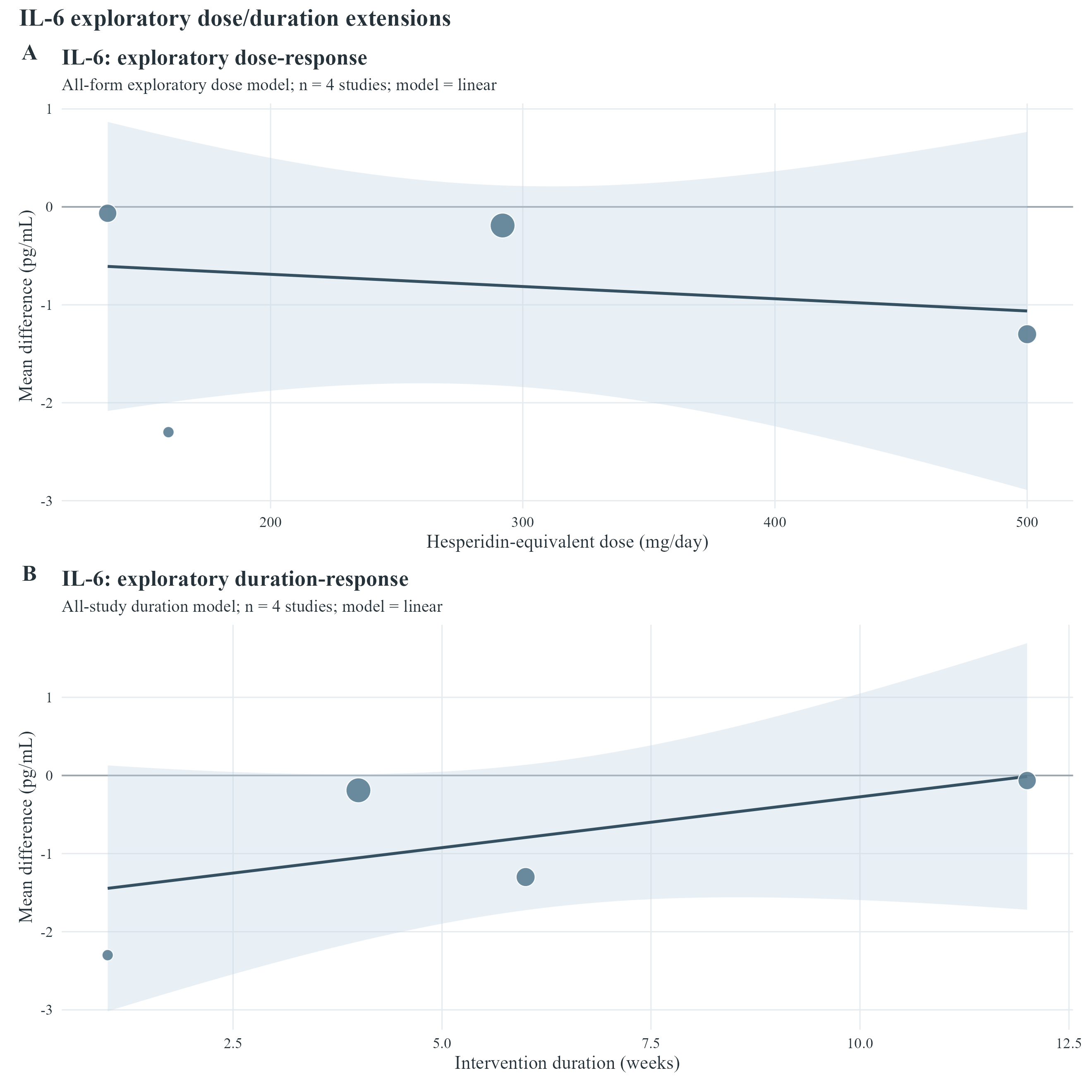


**Supplementary Figure 4**. Exploratory dose- and duration-response analyses of IL-6. Panel A shows the dose-response model, and Panel B shows the duration-response model. Curves represent exploratory meta-regression fits with 95% confidence bands; point size reflects study weight.


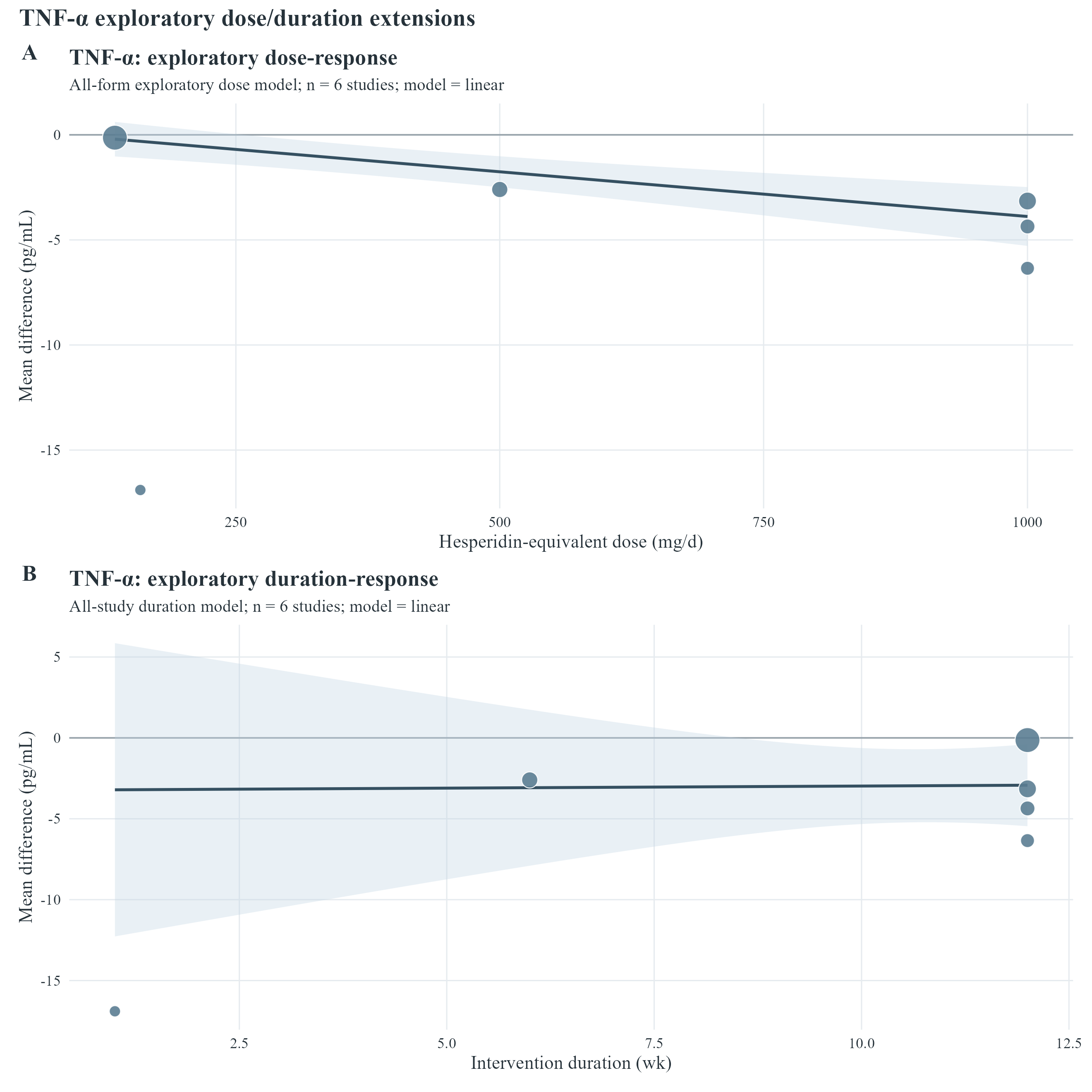
**Supplementary Figure 5**. Exploratory dose- and duration-response analyses of TNF-α. Panel A shows the dose-response model, and Panel B shows the duration-response model. Curves represent exploratory meta-regression fits with 95% confidence bands; point size reflects study weight.

**
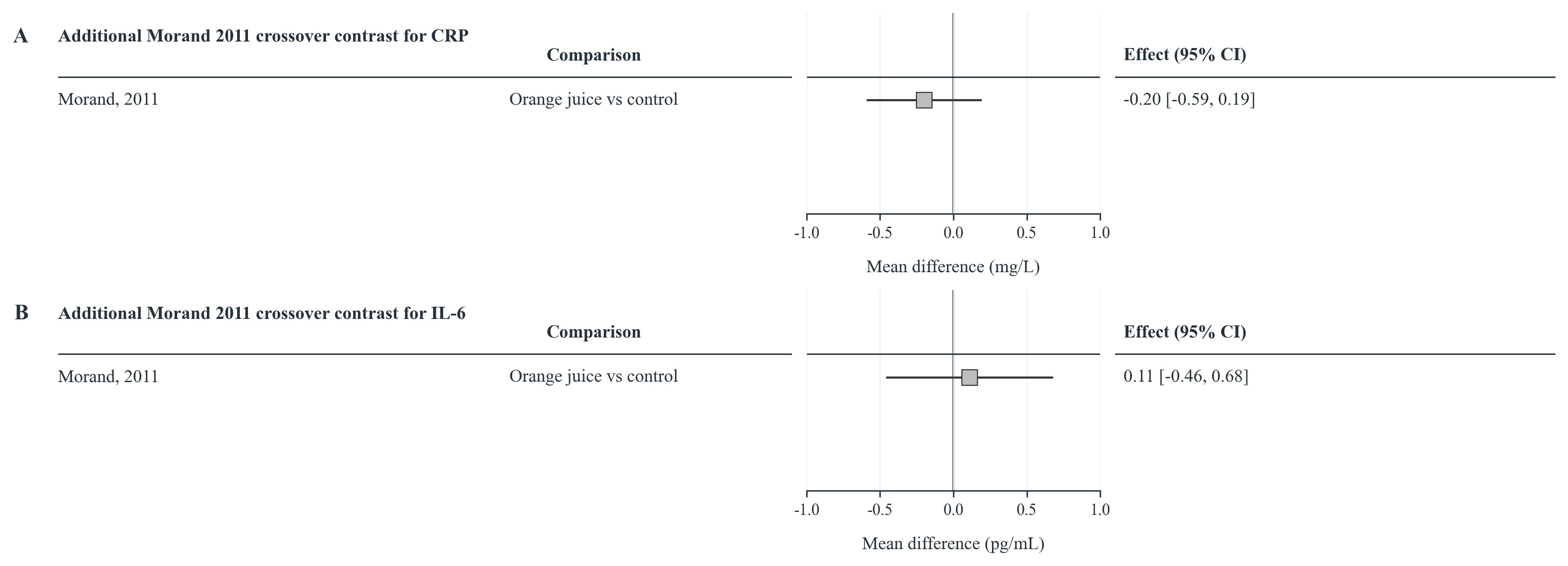
**

**Supplementary Figure 6.** Additional Morand 2011 crossover contrasts. Panel A shows the CRP contrast, and Panel B shows the IL-6 contrast for orange juice versus control. These contrasts are shown separately and are not included in the primary pooled estimates.


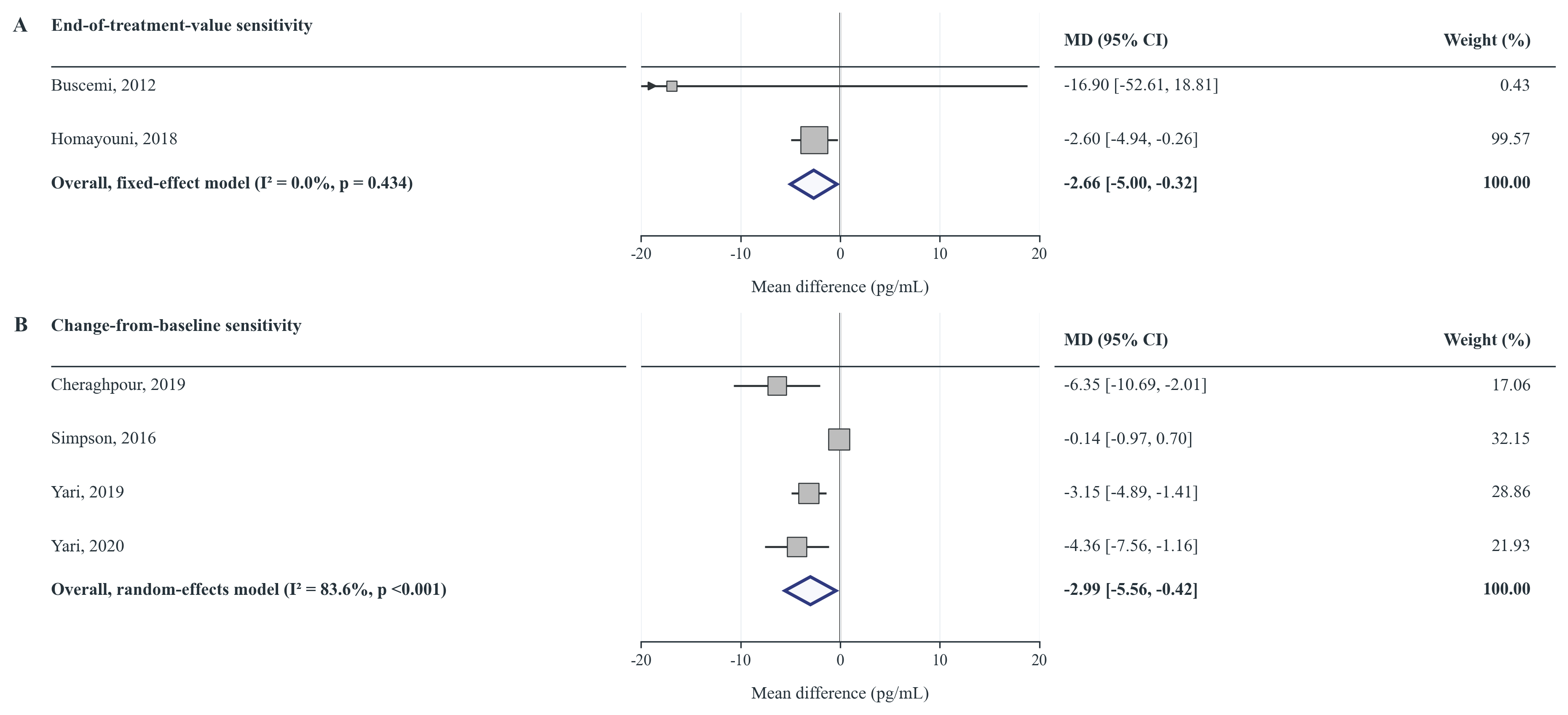


**Supplementary Figure 7**. Supplementary reporting-basis sensitivity plots of TNF-α. Panel A shows the end-of-treatment-value subset, and Panel B shows the change-from-baseline subset. These analyses describe reporting-basis-specific estimates and were not used as the primary sensitivity analysis.


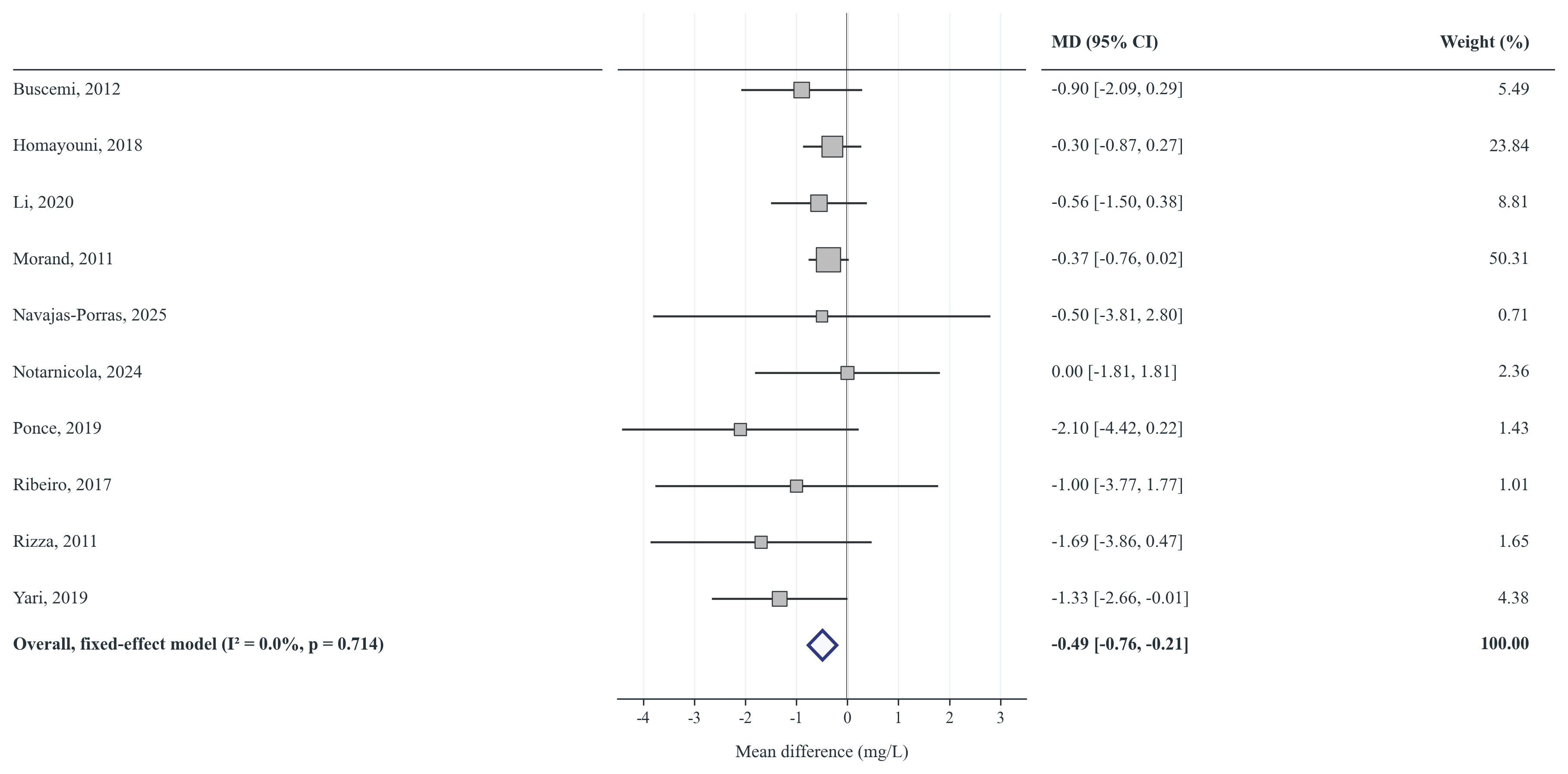


**Supplementary Figure 8**. Supplementary post-only CRP/hsCRP reporting-basis sensitivity plot. This figure describes the end-of-treatment-value subset and was not used as the primary sensitivity analysis.


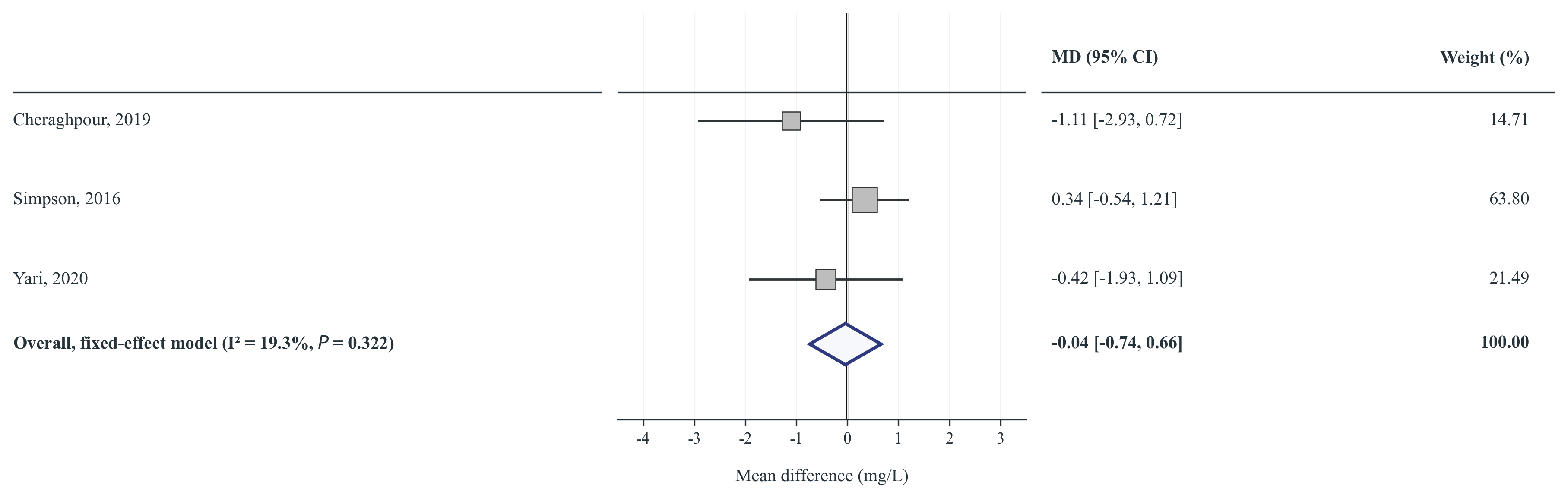


**Supplementary Figure 9**. Supplementary change-from-baseline CRP/hsCRP reporting-basis sensitivity plot. Because this analysis represents a small subset, it is interpreted descriptively.


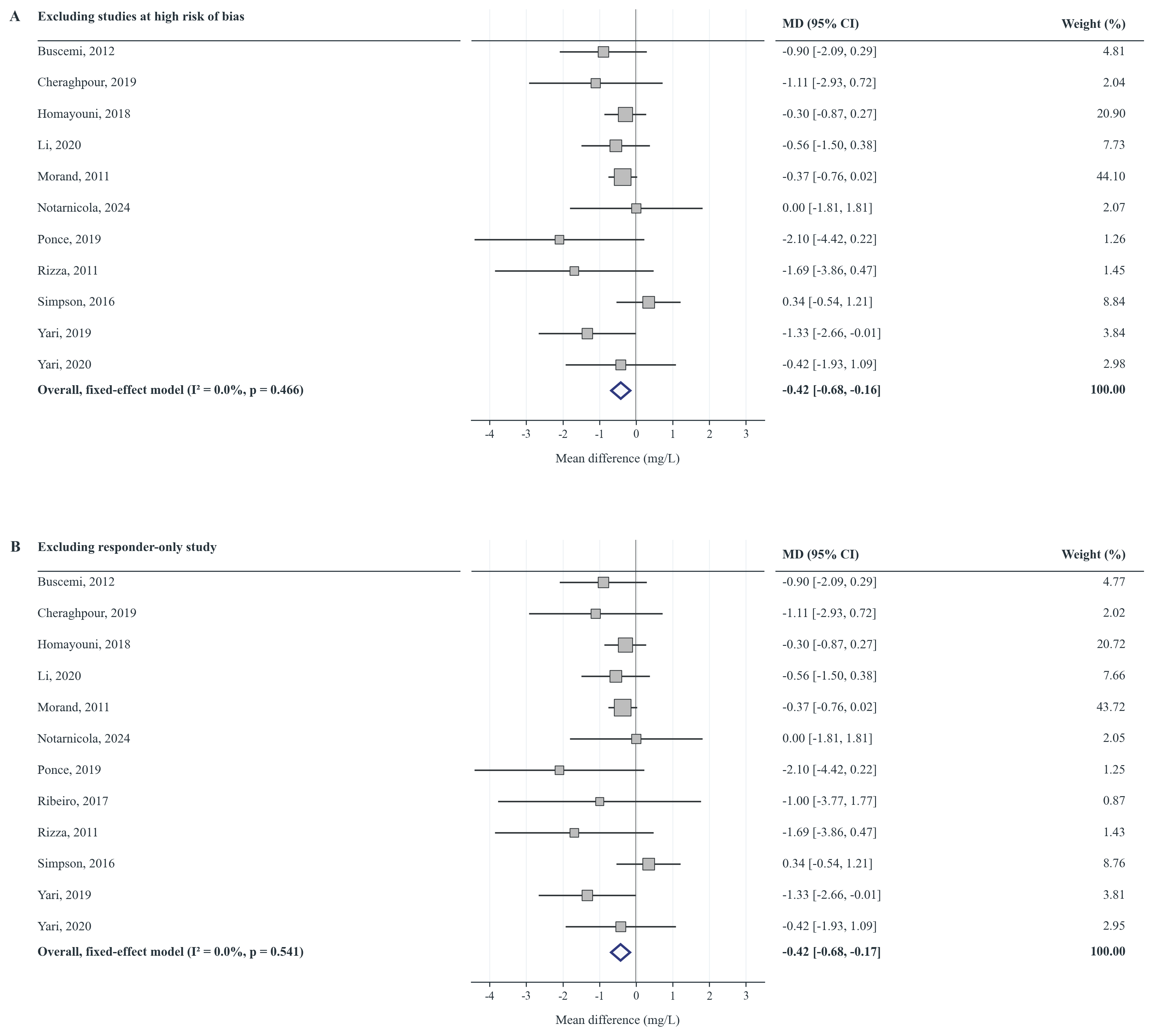

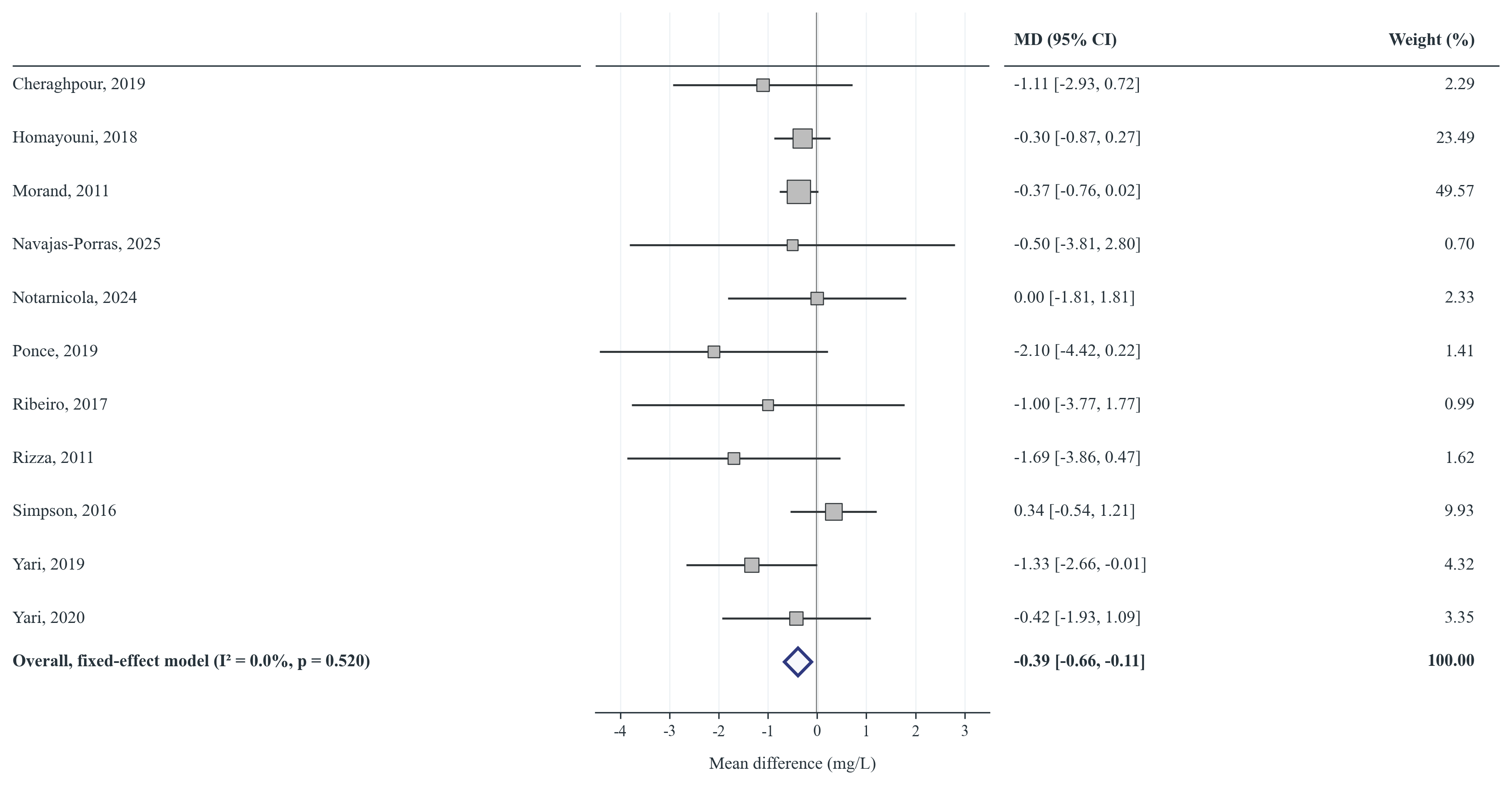
 **Supplementary Figure 10**. Supplementary CRP/hsCRP data-handling sensitivity analysis excluding conservative crossover rows.

**Supplementary Figure 11**. Supplementary CRP/hsCRP exclusion sensitivity analyses. Panel A excludes studies at high risk of bias, and Panel B excludes the responder-only study. The high risk-of-bias exclusion corresponds to the main risk-of-bias sensitivity analysis; the responder-only exclusion is interpreted descriptively.


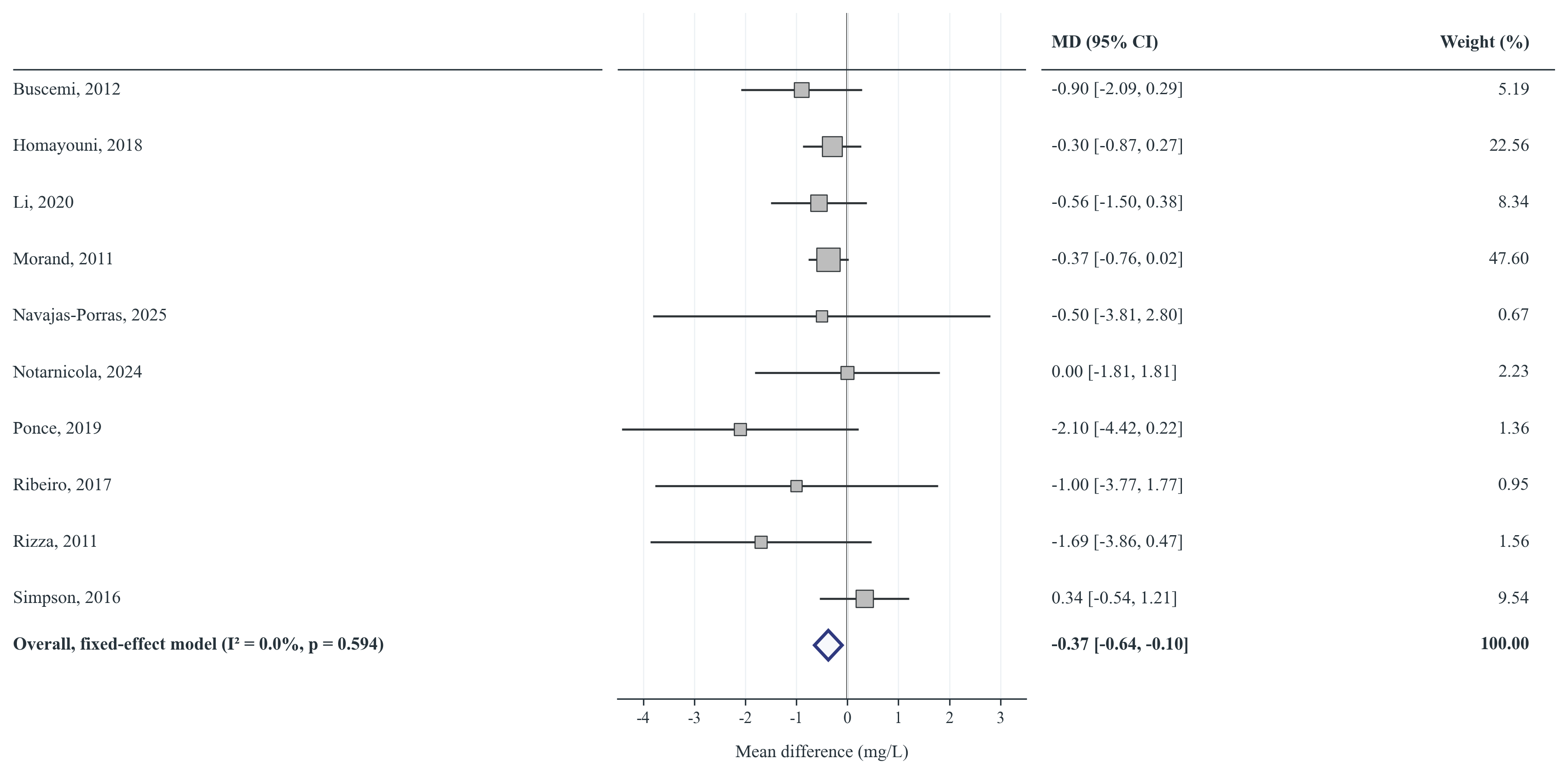


**Supplementary Figure 12**. Supplementary CRP/hsCRP data-handling sensitivity analysis excluding unit-corrected studies.


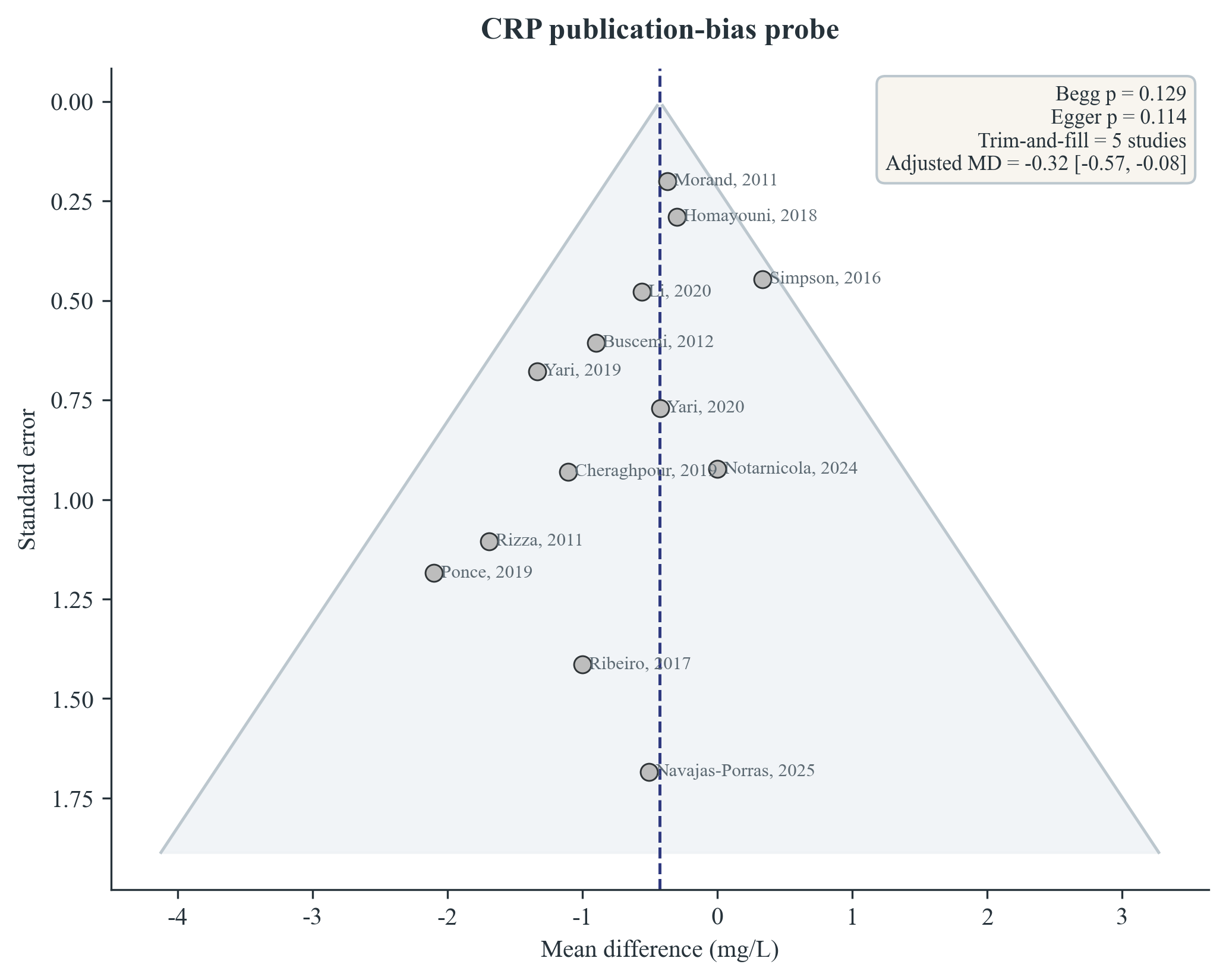
 **Supplementary Figure 13**. Funnel plot of CRP/hsCRP for publication-bias assessment. The vertical dashed line indicates the pooled estimate, the shaded region indicates the pseudo-95% confidence region, and the inset summarizes Begg’s test, Egger’s test, trim-and-fill results, and the adjusted estimate.

**2. Supplementary Tables**

**Supplementary Table 1.** Search strategy.

| Database | Search strategy |
| --- | --- |
| PubMed (77 records) | Search(((((((Hesperidin[MeSH Terms]) OR (Hesperitin[Title/Abstract])) OR (citrus flavonoid[Title/Abstract])) OR (orange juice[Title/Abstract])) OR (orange polyphenols[Title/Abstract])) OR (citrus flavanones[Title/Abstract])) AND ((((((((((((Overweight[MeSH Terms]) OR (Obesity[MeSH Terms])) OR (Body Mass Index[MeSH Terms])) OR (overweight[Title/Abstract])) OR (obese[Title/Abstract])) OR (obesity[Title/Abstract])) OR (weight gain[Title/Abstract])) OR (weight loss[Title/Abstract])) OR (adiposity[Title/Abstract])) OR (metabolic syndrome[Title/Abstract])) OR (diabetes mellitus, type 2[Title/Abstract])) OR (type 2 diabetes[Title/Abstract]))) AND (((((((randomized controlled trial[Publication Type]) OR (randomized[Title/Abstract])) OR (placebo[Title/Abstract])) OR (random[Title/Abstract])) OR (clinical trial[Title/Abstract])) OR (trial[Title/Abstract])) OR (RCT[Title/Abstract]))) |
| Web of Science (367 records) | TS=(Hesperidin OR Hesperitin OR citrus flavonoid OR orange juice OR orange polyphenols OR citrus flavanones) AND TS=(Overweight OR Obesity OR Body Mass Index OR obese OR weight gain OR weight loss OR adiposity OR metabolic syndrome OR diabetes mellitus, type 2 OR type 2 diabetes) AND TS=(randomized controlled trial OR randomized OR placebo OR random OR clinical trial OR trial OR RCT) |
| Scopus (261 records) | ( TITLE-ABS-KEY ( "Hesperidin" OR "Hesperitin" OR "citrus flavonoid" OR "orange juice" OR "orange polyphenols" OR "citrus flavanones" ) AND TITLE-ABS-KEY ( "Overweight" OR "Obesity" OR "Body Mass Index" OR "obese" OR "weight gain" OR "weight loss" OR "adiposity" OR "metabolic syndrome" OR "diabetes mellitus, type 2" OR "type 2 diabetes" ) AND TITLE-ABS-KEY ( "randomized controlled trial" OR "randomized" OR "placebo" OR "random" OR "clinical trial" OR "trial" OR "RCT" ) ) |
| Cochrane Library (174 records) | ((Hesperidin):ab,ti,kw OR (Hesperitin):ab,ti,kw OR (citrus flavonoid):ab,ti,kw OR (orange juice):ab,ti,kw OR (orange polyphenols):ab,ti,kw OR (citrus flavanones):ab,ti,kw) AND ((Overweight):ab,ti,kw OR (Obesity):ab,ti,kw OR (Body Mass Index):ab,ti,kw OR (obese):ab,ti,kw OR (weight gain):ab,ti,kw OR (weight loss):ab,ti,kw OR (adiposity):ab,ti,kw OR (metabolic syndrome):ab,ti,kw OR (diabetes mellitus, type 2):ab,ti,kw OR (type 2 diabetes):ab,ti,kw) AND ((randomized controlled trial):ab,ti,kw OR (randomized):ab,ti,kw OR (placebo):ab,ti,kw OR (random):ab,ti,kw OR (clinical trial):ab,ti,kw OR (trial):ab,ti,kw OR (RCT):ab,ti,kw) |
| Embase (141 records) | ('Hesperidin':ab,ti OR 'Hesperitin':ab,ti OR 'citrus flavonoid':ab,ti OR 'orange juice':ab,ti OR 'orange polyphenols':ab,ti OR 'citrus flavanones':ab,ti) AND ('Overweight':ab,ti OR 'Obesity':ab,ti OR 'Body Mass Index':ab,ti OR 'obese':ab,ti OR 'weight gain':ab,ti OR 'weight loss':ab,ti OR 'adiposity':ab,ti OR 'metabolic syndrome':ab,ti OR 'diabetes mellitus, type 2':ab,ti OR 'type 2 diabetes':ab,ti) AND ('randomized controlled trial':ab,ti OR 'randomized':ab,ti OR 'placebo':ab,ti OR 'random':ab,ti OR 'clinical trial':ab,ti OR 'trial':ab,ti OR 'RCT':ab,ti) |

**Supplementary Table 2.** Excluded trials and reasons for exclusion.

| **Excluded trials** | **Reason for exclusion** |
| --- | --- |
| Osama 2023(1) | This randomized trial did not report the outcomes of interest. |
| Sweidan 2015(2) | This randomized trial did not report the outcomes of interest. |
| Yari 2021(3) | This randomized trial did not report the outcomes of interest. |
| Cesar 2025(4) | This clinical trial intervention measure does not meet the inclusion criteria. |
| Escudero 2018(5) | This clinical trial intervention measure does not meet the inclusion criteria. |
| Unretrievable records identified only by trial number (Irct2012100210181N)(6) | This clinical trial intervention measure does not meet the inclusion criteria. |
| Silveira 2015(7) | This clinical trial intervention measure does not meet the inclusion criteria. |
| Duplicate report of the same study (NCT00983086) (8) | This record is derived from the same clinical trial as an already included publication; excluded to avoid duplicate data. |
| Duplicate report of the same study (NCT02610491) (9) | This record is derived from the same clinical trial as an already included publication; excluded to avoid duplicate data. |
| Duplicate report of the same study (NCT02914249)(10) | This record is derived from the same clinical trial as an already included publication; excluded to avoid duplicate data. |
| Duplicate report of the same study (NCT03734835)(11) | This record is derived from the same clinical trial as an already included publication; excluded to avoid duplicate data. |
| Duplicate report of the same study (NCT02195934) (12) | This record is derived from the same clinical trial as an already included publication; excluded to avoid duplicate data. |
| Homayouni 2017(13) | This record is derived from the same clinical trial as an already included publication; excluded to avoid duplicate data. |
| Bonina 2002(14) | The control group in this clinical trial failed to meet the inclusion criteria and the dosage of hesperidin has not been clearly reported. |
| Nobile 2022(15) | The dosage of hesperidin has not been clearly reported. |
| Cesar 2020(16) | This is a conference abstract. |
| Eghtesadi 2017(17) | This is a conference abstract. |
| Li 2018(18) | This is a conference abstract. |
| Rangel 2013(19) | This is a conference abstract. |
| Rangel 2013(20) | This is a conference abstract. |
| Unretrievable records identified only by trial number (IRCT201407242602N12)(21) | Only the trial registration number for this study was identified; the full text could not be accessed. |
| Unretrievable records identified only by trial number(NCT06680635)(22) | Only the trial registration number for this study was identified; the full text could not be accessed. |
| Mohammad 2016(23) | Full text is in Persian, making it impossible to accurately extract study data and outcomes. |

**References**

1. Osama H, Hamed EO, Mahmoud MA, Abdelrahim MEA. The Effect of Hesperidin and Diosmin Individually or in Combination on Metabolic Profile and Neuropathy among Diabetic Patients with Metabolic Syndrome: ARandomized Controlled Trial. Journal of dietary supplements. 2023;20(5):749-62.

2. Sweidan AMAZ. Bioavailability of citrus flavanones and their effect on cardiovascular health biomarkers. 2015.

3. Yari Z, Cheraghpour M, Hekmatdoost A. Flaxseed and/or hesperidin supplementation in metabolic syndrome: an open-labeled randomized controlled trial. EUROPEAN JOURNAL OF NUTRITION. 2021;60(1):287-98.

4. Cesar T, Oliveira MR, Sandrim V, Mendes A, Bruder R, Oliveira R, et al. Citrus flavonoid supplement enhances glycemic and metabolic control in prediabetic patients on metformin: a randomized controlled trial. FRONTIERS IN NUTRITION. 2025;12.

5. Escudero-Lopez B, Ortega A, Cerrillo I, Rodriguez-Grinolo M-R, Munoz-Hernandez R, Macher HC, et al. Consumption of orange fermented beverage improves antioxidant status and reduces peroxidation lipid and inflammatory markers in healthy humans. JOURNAL OF THE SCIENCE OF FOOD AND AGRICULTURE. 2018;98(7):2777-86.

6. Irct2012100210181N. Evaluation of the effect of Hesperidin supplementation in patients with myocardial infarction. <https://trialsearchwhoint/Trial2aspx?TrialID=IRCT2012100210181N2>. 2013.

7. Silveira JQ, Dourado GKZS, Cesar TB. Red-fleshed sweet orange juice improves the risk factors for metabolic syndrome. International Journal of Food Sciences and Nutrition. 2015;66(7):830-6.

8. Effects of Consumption of Orange Juice on Vascular Protection and Immune Function: Clinical Study on the Specific Contribution of Citrus Flavanones. 2009.

9. The Effect of Hesperidin Administration on Glucose / Insulin Metabolism. 2015.

10. Low-calorie Diet Combined With Orange Juice Results in Weight Loss: Randomized Controlled Trial. 2016.

11. The Effect of Supplementation of Flaxseed, Hesperidin, Flaxseed and Hesperidin Together in Metabolic Syndrome and Its Components: A Randomized, Controlled Study. 2018.

12. Nct. The Orange Juice and Cardiovascular Disease Study. <https://clinicaltrialsgov/show/NCT02195934>. 2014.

13. Homayouni F, Haidari F, Hedayati M, Zakerkish M, Ahmadi K. Hesperidin Supplementation Alleviates Oxidative DNA Damage and Lipid Peroxidation in Type 2 Diabetes: A Randomized Double-Blind Placebo-Controlled Clinical Trial. PHYTOTHERAPY RESEARCH. 2017;31(10):1539-45.

14. Bonina FP, Leotta C, Scalia G, Puglia C, Trombetta D, Tringali G, et al. Evaluation of oxidative stress in diabetic patients after supplementation with a standardised red orange extract. Diabetes, Nutrition and Metabolism - Clinical and Experimental. 2002;15(1):14-9.

15. Nobile V, Pisati M, Cestone E, Insolia V, Zaccaria V, Malfa GA. Antioxidant Efficacy of a Standardized Red Orange (Citrus sinensis (L.) Osbeck) Extract in Elderly Subjects: A Randomized, Double Blind, Controlled Study. NUTRIENTS. 2022;14(20).

16. Cesar T, Benassi R, Ponce O, Nasser M. Orange juice combined to a healthy-eating pattern improved endothelial function and reduced global risk of CHD in metabolic syndrome patients. Proceedings of the Nutrition Society. 2020;79(OCE2).

17. Eghtesadi S, Mohammadi M, Vafa M, Khademhaghighian H, Heidari I, Salehi M, et al. Effects of hesperidin supplementation on weight , glycemic control, lipid profile and infammatory factors in patients with type 2 diabetes: A randomized, controlled clinical trial. Obesity Facts. 2017;10:176.

18. Li L, Birch KM, Boesch C. Effects of blood orange juice consumption on vascular function in healthy overweight subjects of European origin. PROCEEDINGS OF THE NUTRITION SOCIETY. 2018;77(OCE4):E211-E.

19. Rangel O, Rico M, Vallejo F, Boza J, Kellerhals M, Perez de la Cruz A, et al. Consumption of a polyphenol-rich orange juice improves endothelial biomarkers in overweight and obese adults(bionaos study). Annals of Nutrition and Metabolism. 2013;63:213.

20. Rangel OD, Rico MC, Vallejo F, Boza JJ, Kellerhals M, Pérez de La Cruz AJ, et al. Evolution of plasma inflammatory biomarkers after the intake of an orange-based beverage enriched with polyphenols in overweight adults (BIONAOS Study). Proceedings of the Nutrition Society. 2013;72:E77.

21. Irct201407242602N. The effect of hesperidin in the treatment of diabetes. <https://trialsearchwhoint/Trial2aspx?TrialID=IRCT201407242602N12>. 2014.

22. Nct. Effect of Citrus Flavonoids on Obesity. <https://clinicaltrialsgov/ct2/show/NCT06680635>. 2024.

23. Mohammad M, Shahryar E, Reza VM, Iraj H, Masoud S, Esmat S, et al. THE EFFECT OF HESPERIDIN SUPPLEMENTATION ON INDICES OF GLUCOSE AND LIPID, INSULIN LEVELS AND INSULIN RESISTANCE IN PATIENTS WITH TYPE 2 DIABETES: A RANDOMIZED DOUBLE-BLIND CLINICAL TRIAL. Razi Journal of Medical Sciences. 2016;23(143):71-80.

**Supplementary Table 3**. Subgroup analyses of CRP/hsCRP, TNF-α, and IL-6.

## CRP / hsCRP

| Subgroup family | Subgroup | Studies (k) | Primary model | Effect estimate | Within-subgroup heterogeneity | Between-subgroup interaction *P* | Interpretation |
| --- | --- | --- | --- | --- | --- | --- | --- |
| Study type/design | crossover | 4 | fixed | MD -0.47 mg/L (95% CI -0.81 to -0.13) | I² = 0.0%; Q-test *P* = 0.5701 | 0.6826 | No interaction signal |
| Study type/design | parallel | 9 | fixed | MD -0.36 mg/L (95% CI -0.76 to 0.03) | I² = 12.8%; Q-test *P* = 0.4625 | 0.6826 | No interaction signal |
| Intervention type | purified hesperidin | 6 | fixed | MD -0.45 mg/L (95% CI -0.75 to -0.15) | I² = 0.0%; Q-test *P* = 0.5658 | 0.7809 | No interaction signal |
| Intervention type | hesperidin-containing complexes | 7 | fixed | MD -0.36 mg/L (95% CI -0.87 to 0.15) | I² = 18.0%; Q-test *P* = 0.4319 | 0.7809 | No interaction signal |
| Duration | <=6 wk | 7 | fixed | MD -0.42 mg/L (95% CI -0.70 to -0.13) | I² = 0.0%; Q-test *P* = 0.8707 | 0.8883 | No interaction signal |
| Duration | >6 wk | 6 | random | MD -0.68 mg/L (95% CI -1.50 to 0.13) | I² = 37.9%; Q-test *P* = 0.1934 | 0.8883 | No interaction signal |
| Dose | <=500 mg/d | 10 | fixed | MD -0.37 mg/L (95% CI -0.64 to -0.10) | I² = 0.0%; Q-test *P* = 0.5944 | 0.1976 | No interaction signal |
| Dose | >500 mg/d | 3 | fixed | MD -0.97 mg/L (95% CI -1.85 to -0.10) | I² = 0.0%; Q-test *P* = 0.6651 | 0.1976 | No interaction signal |
| Health status | otherwise healthy or obesity only | 3 | fixed | MD -0.40 mg/L (95% CI -0.76 to -0.04) | I² = 0.0%; Q-test *P* = 0.9331 | 0.8394 | No interaction signal |
| Health status | cardiometabolic disease/risk phenotype | 10 | fixed | MD -0.45 mg/L (95% CI -0.82 to -0.08) | I² = 20.0%; Q-test *P* = 0.3747 | 0.8394 | No interaction signal |
| Lifestyle cointervention | active diet/lifestyle cointervention | 6 | fixed | MD -1.07 mg/L (95% CI -1.84 to -0.31) | I² = 0.0%; Q-test *P* = 0.8848 | 0.0772 | No interaction signal |
| Lifestyle cointervention | no active lifestyle cointervention | 7 | fixed | MD -0.34 mg/L (95% CI -0.62 to -0.07) | I² = 0.1%; Q-test *P* = 0.5397 | 0.0772 | No interaction signal |
| Risk of bias | low risk of bias | 3 | fixed | MD -0.45 mg/L (95% CI -0.98 to 0.08) | I² = 17.8%; Q-test *P* = 0.3631 | 0.9171 | No interaction signal |
| Risk of bias | some concerns/high risk of bias | 10 | fixed | MD -0.42 mg/L (95% CI -0.71 to -0.12) | I² = 0.0%; Q-test *P* = 0.5494 | 0.9171 | No interaction signal |

## TNF-α

| Subgroup family | Subgroup | Studies (k) | Primary model | Effect estimate | Within-subgroup heterogeneity | Between-subgroup interaction *P* | Interpretation |
| --- | --- | --- | --- | --- | --- | --- | --- |
| Study type/design | crossover | 1 | single | MD -16.90 pg/mL (95% CI -52.61 to 18.81) | Not applicable | Descriptive only because at least one subgroup level had k < 2 | Descriptive only |
| Study type/design | parallel | 5 | random | MD -2.79 pg/mL (95% CI -4.76 to -0.82) | I² = 77.6%; Q-test *P* = 0.0003 | Descriptive only because at least one subgroup level had k < 2 | Descriptive only |
| Intervention type | purified hesperidin | 4 | fixed | MD -3.43 pg/mL (95% CI -4.66 to -2.20) | I² = 0.0%; Q-test *P* = 0.4502 | <0.001 | Interaction signal; interpret cautiously |
| Intervention type | hesperidin-containing complexes | 2 | fixed | MD -0.15 pg/mL (95% CI -0.98 to 0.69) | I² = 0.0%; Q-test *P* = 0.3577 | <0.001 | Interaction signal; interpret cautiously |
| Duration | <=6 wk | 2 | fixed | MD -2.66 pg/mL (95% CI -5.00 to -0.32) | I² = 0.0%; Q-test *P* = 0.4336 | 0.9673 | No interaction signal |
| Duration | >6 wk | 4 | random | MD -2.99 pg/mL (95% CI -5.56 to -0.42) | I² = 83.6%; Q-test *P* = 0.0002 | 0.9673 | No interaction signal |
| Dose | <=500 mg/d | 3 | random | MD -1.19 pg/mL (95% CI -3.57 to 1.19) | I² = 58.8%; Q-test *P* = 0.1013 | 0.0386 | Interaction signal; interpret cautiously |
| Dose | >500 mg/d | 3 | fixed | MD -3.75 pg/mL (95% CI -5.19 to -2.31) | I² = 2.8%; Q-test *P* = 0.3732 | 0.0386 | Interaction signal; interpret cautiously |
| Health status | cardiometabolic disease/risk phenotype | 6 | random | MD -2.84 pg/mL (95% CI -4.81 to -0.86) | I² = 73.7%; Q-test *P* = 0.0005 | Not estimable because only one subgroup level was represented | Descriptive only |
| Lifestyle cointervention | active diet/lifestyle cointervention | 3 | fixed | MD -3.75 pg/mL (95% CI -5.19 to -2.31) | I² = 2.8%; Q-test *P* = 0.3732 | 0.0386 | Interaction signal; interpret cautiously |
| Lifestyle cointervention | no active lifestyle cointervention | 3 | random | MD -1.19 pg/mL (95% CI -3.57 to 1.19) | I² = 58.8%; Q-test *P* = 0.1013 | 0.0386 | Interaction signal; interpret cautiously |
| Risk of bias | low risk of bias | 2 | fixed | MD -3.45 pg/mL (95% CI -5.51 to -1.38) | I² = 54.9%; Q-test *P* = 0.1364 | 0.4488 | No interaction signal |
| Risk of bias | some concerns/high risk of bias | 4 | random | MD -2.32 pg/mL (95% CI -4.86 to 0.21) | I² = 78.1%; Q-test *P* = 0.0020 | 0.4488 | No interaction signal |

## IL-6

| Subgroup family | Subgroup | Studies (k) | Primary model | Effect estimate | Within-subgroup heterogeneity | Between-subgroup interaction *P* | Interpretation |
| --- | --- | --- | --- | --- | --- | --- | --- |
| Study type/design | crossover | 2 | random | MD -1.05 pg/mL (95% CI -3.09 to 0.98) | I² = 78.0%; Q-test *P* = 0.0330 | 0.8138 | No interaction signal |
| Study type/design | parallel | 2 | random | MD -0.70 pg/mL (95% CI -1.91 to 0.51) | I² = 69.5%; Q-test *P* = 0.0703 | 0.8138 | No interaction signal |
| Intervention type | purified hesperidin | 2 | random | MD -0.69 pg/mL (95% CI -1.77 to 0.40) | I² = 75.6%; Q-test *P* = 0.0428 | 0.8355 | No interaction signal |
| Intervention type | hesperidin-containing complexes | 2 | random | MD -1.04 pg/mL (95% CI -3.21 to 1.13) | I² = 77.1%; Q-test *P* = 0.0367 | 0.8355 | No interaction signal |
| Duration | <=6 wk | 3 | random | MD -1.03 pg/mL (95% CI -2.15 to 0.09) | I² = 73.8%; Q-test *P* = 0.0236 | Descriptive only because at least one subgroup level had k < 2 | Descriptive only |
| Duration | >6 wk | 1 | single | MD -0.06 pg/mL (95% CI -1.04 to 0.91) | Not applicable | Descriptive only because at least one subgroup level had k < 2 | Descriptive only |
| Dose | <=500 mg/d | 4 | random | MD -0.74 pg/mL (95% CI -1.57 to 0.09) | I² = 66.7%; Q-test *P* = 0.0371 | Not estimable because only one subgroup level was represented | Descriptive only |
| Health status | otherwise healthy or obesity only | 1 | single | MD -0.19 pg/mL (95% CI -0.76 to 0.38) | Not applicable | Descriptive only because at least one subgroup level had k < 2 | Descriptive only |
| Health status | cardiometabolic disease/risk phenotype | 3 | random | MD -1.05 pg/mL (95% CI -2.22 to 0.11) | I² = 66.3%; Q-test *P* = 0.0571 | Descriptive only because at least one subgroup level had k < 2 | Descriptive only |
| Lifestyle cointervention | no active lifestyle cointervention | 4 | random | MD -0.74 pg/mL (95% CI -1.57 to 0.09) | I² = 66.7%; Q-test *P* = 0.0371 | Not estimable because only one subgroup level was represented | Descriptive only |
| Risk of bias | low risk of bias | 1 | single | MD -1.30 pg/mL (95% CI -2.21 to -0.39) | Not applicable | Descriptive only because at least one subgroup level had k < 2 | Descriptive only |
| Risk of bias | some concerns/high risk of bias | 3 | random | MD -0.55 pg/mL (95% CI -1.55 to 0.46) | I² = 65.5%; Q-test *P* = 0.0891 | Descriptive only because at least one subgroup level had k < 2 | Descriptive only |

**Supplementary Table 4.** Exploratory dose- and duration-response analyses.

| Analysis layer | Dataset | Studies (k) | Model | Main result | Interpretation |
| --- | --- | --- | --- | --- | --- |
| Dose-response | CRP / hsCRP, all form-specific interventions with interpretable dose values | 11 | quadratic exploratory meta-regression | No clear linear or non-linear dose signal (linear coefficient p = 0.975; quadratic coefficient p = 0.737) | Exploratory result |
| Duration-response | CRP / hsCRP, all studies | 13 | quadratic exploratory meta-regression | No clear duration signal (linear coefficient p = 0.305; quadratic coefficient p = 0.317) | Exploratory result |
| Dose-response | CRP / hsCRP, capsule-only subset | 6 | quadratic exploratory meta-regression | No clear capsule-only dose signal (linear coefficient p = 0.791; quadratic coefficient p = 0.643) | Exploratory result |
| Dose-response | IL-6, all studies with interpretable dose values | 4 | linear exploratory meta-regression | No clear dose signal (coefficient p = 0.730) | Exploratory result |
| Duration-response | IL-6, all studies | 4 | linear exploratory meta-regression | No clear duration signal (coefficient p = 0.299) | Exploratory result |
| Dose-response | TNF-α, all studies with interpretable dose values | 6 | linear exploratory meta-regression | A larger reduction was observed at higher doses (coefficient p < 0.001) | Exploratory result; interpret cautiously |
| Duration-response | TNF-α, all studies | 6 | linear exploratory meta-regression | No clear duration signal (coefficient p = 0.955) | Exploratory result |

**Notes:**

- These extensions should be interpreted as exploratory analyses rather than as part of the primary inferential analysis.
- Studies without directly interpretable daily hesperidin exposure values were not included in the continuous dose models.
- Mixed capsule and food-based interventions continue to limit causal dose-response interpretation; these models do not support matrix-specific clinical recommendations.
- The p values in this table are exploratory meta-regression coefficient tests, not primary pooled-effect or heterogeneity tests.

**Supplementary Table 5.** Sensitivity analyses, including leave-one-out diagnostics and exclusions of studies at high risk of bias; reporting-basis and data-handling checks are provided as supplementary analyses.

| Outcome | Sensitivity set | Studies (k) | Model / analysis basis | Effect estimate | Heterogeneity / test | Main reading |
| --- | --- | --- | --- | --- | --- | --- |
| CRP / hsCRP | Primary pooled reference | 13 | Fixed-effect MD, mixed end-of-treatment and change-from-baseline basis | MD -0.4254 mg/L (95% CI -0.6838 to -0.1670) | I² = 0.0%; Q-test p = 0.626; overall-effect p = 0.001 | Primary CRP/hsCRP estimate; most stable signal in the evidence base |
| CRP / hsCRP | Leave-one-out diagnostics | 12 after each omission | Fixed-effect MD | Recalculated MDs ranged from -0.4981 to -0.3897 mg/L; all 95% CIs excluded the null | I² = 0.0% in all omissions | Direction and statistical interpretation remained stable after omitting any single study |
| CRP / hsCRP | Excluding studies at high risk of bias (Navajas-Porras 2025 and Ribeiro 2017) | 11 | Fixed-effect MD | MD -0.4199 mg/L (95% CI -0.6802 to -0.1595) | I² = 0.0%; Q-test p = 0.466; overall-effect p = 0.002 | Main CRP/hsCRP interpretation was unchanged |
| IL-6 | Primary pooled reference | 4 | Random-effects MD | MD -0.74 pg/mL (95% CI -1.57 to 0.09) | I² = 66.7%; Q-test p = 0.037; overall-effect p = 0.081 | Supportive inflammatory outcome remained uncertain |
| IL-6 | Leave-one-out diagnostics | 3 after each omission | Random-effects MD | Recalculated MDs ranged from -1.05 to -0.49 pg/mL; all 95% CIs crossed the null | I² = 58.3% to 73.8% | Uncertainty persisted after each single-study omission |
| IL-6 | Excluding studies at high risk of bias | Not applicable | RoB check | No high risk-of-bias study entered this outcome pool | Not applicable | No high risk-of-bias exclusion was performed for IL-6 |
| TNF-α | Primary pooled reference | 6 | Random-effects MD | MD -2.8365 pg/mL (95% CI -4.8080 to -0.8650) | I² = 73.7%; Q-test p < 0.001; overall-effect p = 0.005 | Favorable direction, but certainty was very low and heterogeneity was substantial |
| TNF-α | Leave-one-out diagnostics | 5 after each omission | Random-effects MD | Recalculated MDs ranged from -3.45 to -2.29 pg/mL; all 95% CIs remained below the null | I² = 0.0% only after omitting Simpson 2016; otherwise I² = 71.0% to 79.5% | Direction remained favorable, but heterogeneity generally persisted |
| TNF-α | Excluding studies at high risk of bias | Not applicable | RoB check | No high risk-of-bias study entered this outcome pool | Not applicable | No high risk-of-bias exclusion was performed for TNF-α |
| TAC | Primary pooled reference | 3 | Random-effects Hedges’ g across different TAC assays | SMD -0.19 (95% CI -0.78 to 0.40) | I² = 77.6%; Q-test p = 0.010; overall-effect p = 0.530 | Antioxidant-capacity evidence remained very uncertain |
| TAC | Excluding high risk-of-bias Ribeiro 2017 | 2 | Two-study residual Hedges’ g set | SMD -0.49 (95% CI -0.85 to -0.14) | Not emphasized because only two studies remained | Result suggested lower TAC in the intervention group and should not be interpreted as antioxidant benefit |
| MDA | Sensitivity analysis | Not applicable | Single-study outcome | Single-study estimate from Ribeiro 2017: MD -0.10 mM (95% CI -1.36 to 1.16) | No pooled overall-effect or heterogeneity test | Leave-one-out and high risk-of-bias sensitivity analyses were not possible |
